# Multi-motor position synchronization control method based on non-singular fast terminal sliding mode control

**DOI:** 10.1371/journal.pone.0281721

**Published:** 2023-06-15

**Authors:** Chun-Yang Lan, He Wang, Xin Deng, Xu-Feng Zhang, Hua Song

**Affiliations:** 1 School of Mechanical Engineering and Automation, University of Science and Technology Liaoning, Anshan, China; 2 School of Electronics and Information Engineering, University of Science and Technology Liaoning, Anshan, China; Lanzhou University of Technology, CHINA

## Abstract

In order to improve the position high-precision synchronization performance of multi-motor synchronous control, a multi-motor position synchronization control method based on non-singular fast terminal sliding mode control (NFTSMC) combined with an improved deviation coupling control structure (Improved Deviation Coupling Control(IDCC), NFTSMC+IDCC). Firstly, this paper designs a sliding mode controller using a non-singular fast terminal sliding mode surface with a Permanent Magnet Synchronous Motor (PMSM) as the control object. Secondly, the deviation coupling is improved to enhance the coupling between multiple motors and achieve position synchronization. Finally, the simulation results show that the total error of multi-motor position synchronization under NFTSMC control is 0.553r in the simulation of multi-motor synchronization control under the same working conditions, which is 2.873r and 1.772r less than that of SMC and FTSMC in terms of speed error, and the anti-disturbance performance is 83.68% and 76.22% higher than that of both of them, respectively. In the subsequent simulation of the improved multi-motor position synchronization structure, the total error of the multi-motor position is in the range of 0.56r-0.58r at three speeds, which is much smaller than the synchronization error under the Ring Coupling Control (RCC) structure and Deviation Coupling Control (DCC) structure, showing a better The synchronization error is much smaller than that of the RCC structure and DCC structure, which shows better position synchronization performance. Therefore, the multi-motor position synchronization control method proposed in this paper has a good position synchronization effect and achieves the control effect of small displacement error and fast convergence of the multi-motor position synchronization control system after being disturbed, the control performance is significantly improved.

## Introduction

Multi-motor position synchronization occupies a central place in modern industrial development. Currently, most of the articles in the field of multi-motor control focus on the importance of speed synchronization, but with the development of society and the needs of industry, the importance of multi-motor position synchronization is coming to the fore. For example, in rail lifting and multi-axis chain machines, the position of each motor needs to be highly synchronized, so the synchronization of multi-motor motion position is better than the synchronization of speed in these conditions, and multi-motor position synchronization has a wide range of applications in industrial manufacturing. The research history, control algorithms, and structure of multi-motor position synchronization are similar to those of speed synchronization. The single-motor control algorithm dramatically affects the performance of each individual motor, including the starting performance of the motor during operation and the anti-disturbance performance of the motor when receiving disturbances. The multi-motor control algorithm determines the process of the multi-motor system. The most important is the coupling performance between the multi-motors. The better the coupling performance, the smaller the anti-disturbance error between the motors.

The method proposed in this paper can be applied to multi-motor synchronous lifting conditions, such as the process control of 100 m rail lifting. In rail production, rail lifting is significant because lifting is mainly carried out in the open air, so the lifting process is often disturbed by uncertain external factors. Rail lifting needs multi-motor synchronous control, due to the current limited lateral distance of the train carrying rails, in order to prevent the phenomenon of rail legs extruding each other, so for the lifting of rails, multi-motors require high precision position synchronization, and also have to have anti-interference characteristics to maintain a high degree of multi-motor coupling. Since permanent magnet synchronous motor (PMSM) has the advantages of high efficiency and overload resistance to adapt to rail lifting conditions, this paper provides high precision position control for PMSM.

This paragraph presents and analyzes the current research status of multi-motor position synchronization and non-singular fast terminal sliding film control methods. Multi-point position control techniques are currently used more frequently and maturely on multi-axis systems [1–6]. There are some similarities between multi-axis system control and multi-motor control in control techniques and control processes. For example, the cross-coupling control strategies and adaptive robust control methods used in the literature [2, 3] are often used in multi-motor synchronous control, and both are affected by chance factors in the control process. Similarly, in the control process of a robotic arm, the motors of each joint need to be precisely controlled to grasp something at a particular place [7, 8]. In both pieces of literature, sliding film control is used, and it is seen that the sliding film control algorithm occupies a superior position in terms of high accuracy and strong stability in working conditions, so this paper uses a non-singular fast terminal sliding film surface design controller. The use of position synchronization techniques in linear servo systems is also more widespread [9–11]. In the literature [12], a finite-time adaptive neural network (NN) position-tracking control method is proposed, and the simulation results show that the motor tracking performance under the control of this method is better, but the paper does not simulate the anti-disturbance property. Similarly, in the literature [13] no simulation was performed for this chance case. There are more algorithms for the sliding film algorithm, and most of them are currently controlled by combining the observer method [14–19], but the simulation results show poor steady-state performance and the presence of a jitter. And there is room for improvement in the start-up performance and anti-disturbance performance. In the literature [15, 20], an adaptive non-singular fast terminal sliding mode control method is proposed for PMLSM control, and although some overshoot occurs, the control performance is improved compared with other methods. However, there is a lack of simulation in the paper, and there is inadequate validation [21]. The literature [22, 23] shows better control results, and the model in their literature [22] can be used as a reference. These different control methods can be used to control the speed of permanent magnet synchronous motors by combining direct torque control with modern control methods. In the literature [24], in order to solve the speed tracking problem of a permanent magnet synchronous motor (PMSM) under the influence of parameter uncertainties and external load torque disturbances, two robust controllers, backstepping and adaptive backstepping, are designed to drive the speed of the PMSM to a predefined trajectory. From the simulation, it can be seen that in order to verify the immunity of the motor to disturb the motor with load torque and observe the speed variation of the motor. The verification method proposed in this paper is similar to that of this paper in that load disturbance is applied to the motor to verify the motor motion, with the difference that this paper demonstrates the fluctuation of the motor speed while this paper confirms the position coupling between multiple motors after receiving the disturbance. Also, the simulation conditions in this literature are different from this paper. The former verifies the stability of motor speed and torque at low speed and small torque, while the latter demonstrates the positional coupling between multiple motors at high speed and ample torque. The literature [25–27] expands the study based on sliding mode control for the direction of harmonic distortion suppression to improve harmonic compensation. The combination of the approximation-based adaptive fractional sliding mode control (SMC) method and DLRFNN structure in the literature [25] for high-precision management of micro-gyroscopes can reduce the steady-state error of micro-gyroscopes to a smaller fluctuation range to improve the steady-state accuracy and dynamic sensitivity of micro-gyroscopes, which is the research direction of motor-based control in this paper. Multi-motor multipoint control is highly researched and developed in several industries [28–30], and the literature [30] uses a leader-follower scheme in solving the air formation control problem of a networked quad rotor with fixed time stability, which is developed both on the basis of the master-slave multipoint control principle. Also, a fixed-time distributed non-switching non-star formation control protocol (FDNNFCP) is proposed for the position loop of each following aircraft. In addition, a trajectory tracking controller (TTC) is designed for the leader. It not only improves the system’s robustness, but also ensures no steady-state error. Today’s artificial intelligence techniques combine fuzzy neural, sliding film genetic, fuzzy genetic, and genetic algorithms with good performance [31].

**Fig 1:**
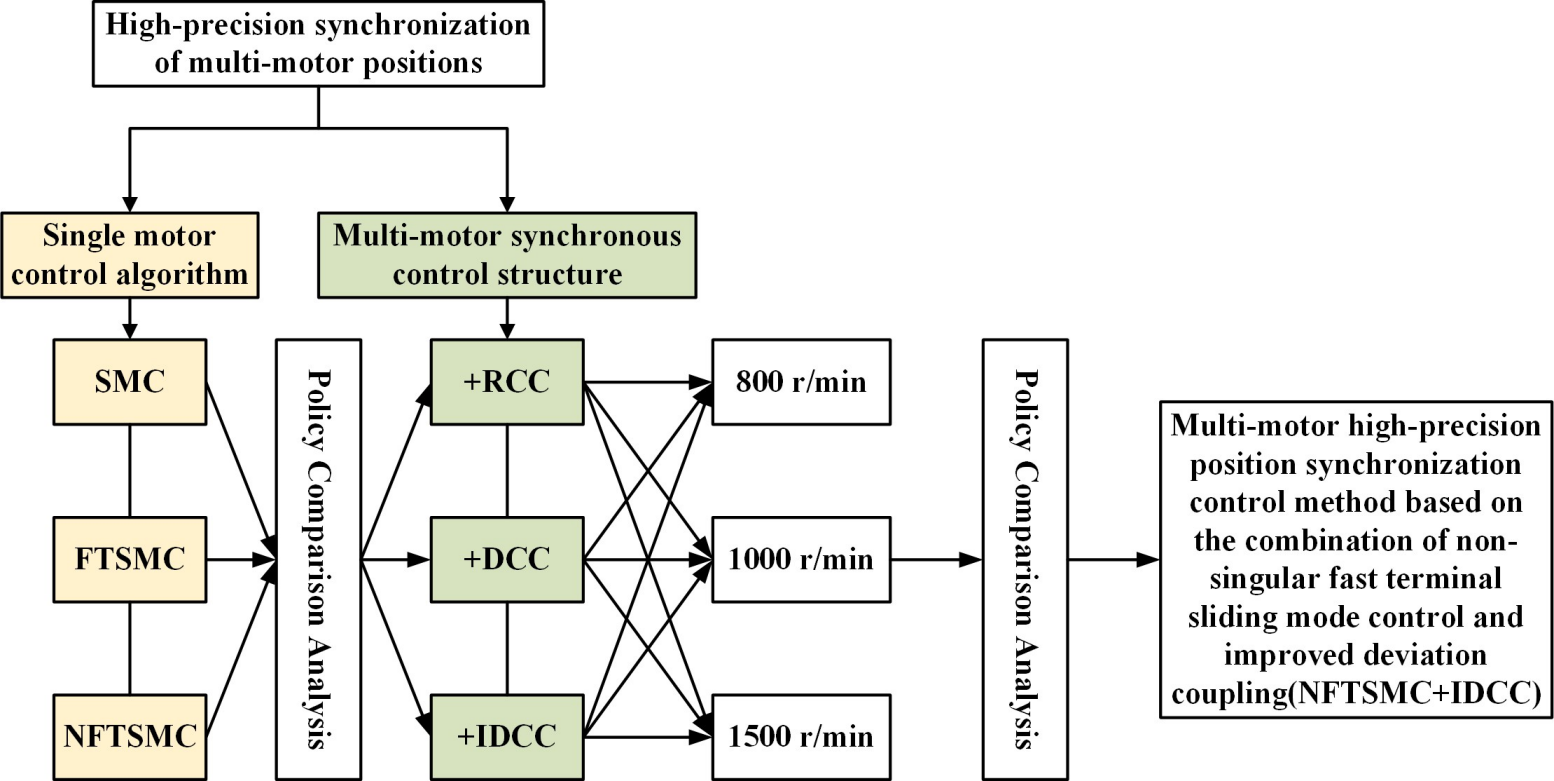
Block diagram of the overall research flow of multi-motor position synchronization.

In this paper, two aspects are considered to improve the multi-motor position synchronization performance. The first aspect is the non-singular fast terminal sliding mode control (NFTSMC) algorithm for the problem of low single-motor immunity to perturbation, and the second aspect is the deviation-coupled control structure as the motor synchronization control strategy for the problem of multi-motor position synchronization, which is improved based on the original control structure. Therefore, this paper proposes a multi-motor position synchronous control method based on non-singular fast terminal sliding mode control, in which NFTSMC can improve the starting capability and anti-disturbance of each motor, and then improve the control quality of the whole multi-motor control system, and the improved deviation coupling enhances the coupling between multi-motors, and the combination of NFTSMC can realize the position high-precision synchronous control between multi-motors. This method combines the advantages of fast start-up and strong anti-disturbance under NFTSMC control with the advantages of strong coupling and displacement signal feedback in the improved deviation coupling control strategy, and such a combination can make it possible to enhance the robustness of the multi-motor position synchronous control system and make the system error converge quickly. In this paper, we take PMSM as the control object and build its model, design the velocity controller using non-singular fast terminal sliding film surface, build the simulation model based on MATLAB/Simulink, and compare the simulation with the results of other control methods and control structures. The specific study steps are shown in Fig 1.

The contributions of this paper are as follows:

1. For the problem of the control algorithm in single motor control, a non-singular fast terminal sliding mode control algorithm is designed, and the speed controller is built in MATLAB/Simulink based on this algorithm, which shortens the speed response time and enhances the anti-disturbance performance of the co-magnet synchronous motor.
2. Since the purpose of this article is multi-motor position synchronization, the speed output in the original deviation-coupled speed control structure is integrated so that its output is position change information, and the input of its original speed compensator structure is changed to the position information of each motor for input. It makes it possible to compensate for the position information in real-time.
3. For the problem of excessive error in multi-motor synchronous control, the original deviation coupling control structure is improved. The improved deviation coupling control structure enhances the coupling between multi-motor systems and reduces the synchronization error.
4. In order to improve the robustness of synchronous control of multiple permanent magnet synchronous motors, a multi-motor speed synchronization control method based on non-singular fast terminal sliding mode and improved deviation coupling is proposed. The effectiveness and superiority of the proposed method are demonstrated by comparing it with the simulation results of other control algorithms and structures.

The following parts of this paper are organized as follows. The second part first establishes the mathematical model of the permanent magnet synchronous motor. Secondly, the design process and stability proof of the non-singular fast terminal sliding mode control method are presented in the third part. The fourth part presents the deviation-coupled multi-motor synchronous control structure and the improvement method. The simulation comparison results of the system are given in the fifth part. Finally, some conclusions are given in the sixth part.

In order to improve the readability of the article that follows, Table 1 is attached for reference.

**Table 1:** Symbol naming table.

symbol	meaning of symbols
*u*_*d*_	d-axis voltage
*u*_*q*_	q-axis voltage
*i*_*d*_	d-axis current
*i*_*q*_	q-axis current
*R*	Stator resistance
*ψ*_*d*_	d-axis magnetic chain
*ψ*_*q*_	q-axis magnetic chain
*ω*_*e*_	Motor angular velocity
*L*_*d*_	d-axis inductor
*L*_*q*_	q-axis inductor
*ψ*_*f*_	Permanent magnet flux linkage
*P*_*n*_	Motor pole pairs
*B*	friction coefficient
*ω*_m_	Mechanical angular velocity
*T*_*L*_	load torque
*T*_*e*_	Electromagnetic torque
*J*	Moment of inertia
*N*_*r*_	motor speed
*c, M*_*u*_ *, q*	SMC controller parameters
*K*_1_ *, K*_2_ *, K*_3_ *, c*_1_ *, c*_2_ *, q, h, p, g,*	FTSMC controller parameters
*ϕ,γ, m, n, c, p*_0_ *, q*_0_ *, β*	NFTSMC controller parameters

### PMSM mathematical model

For the next reasonably convenient construction of the speed controller and overall simulation model, a table-mounted PMSM motor is generally used as an example and the following conditions are assumed to be met when the motor is running.

1. Ignoring the saturation of the stator core, the magnetic circuit characteristics are linear and the parameters of the inductor do not change.
2. Ignoring core eddy currents and hysteresis losses.
3. The electronic windings in the motor are symmetrical to each other and differ by 120 degrees.

The mathematical model based on the *d-q* coordinate system is

$$\left\{\begin{array}{l} u_{d}=Ri_{d}+\frac{d}{dt}\psi_{d}-\omega_{e}\psi_{q}\\ u_{q}=Ri_{q}+\frac{d}{dt}\psi_{q}-\omega_{e}\psi_{d} \end{array}\right.$$ (1)

And the equation of the stator magnetic chain can be written as

$$\left\{\begin{array}{l} \psi_{d}=L_{d}i_{d}+\psi_{f}\\ \psi_{q}=L_{q}i_{q} \end{array}\right.$$ (2)

Bringing (1) into (2), we can get both the stator voltage equation

$$\left\{\begin{array}{l} u_{d}=Ri_{d}+L_{d}\frac{d}{dt}\psi_{d}-w_{e}L_{q}i_{q}\\ u_{q}=Ri_{q}+L_{q}\frac{d}{dt}\psi_{q}+w_{e}\left(L_{q}i_{q}+\psi_{f}\right) \end{array}\right.$$ (3)

Where *u*_*d*_, *u*_*q*_ are the *d-q*-axis components of the stator voltage; *i*_*d*_, *i*_*q*_ are the *d-q*-axis components of the stator current; *R* is the resistance of the stator; *ψ*_*d*_, *ψ*_*q*_ is the *d-q*-axis components of the stator magnetic chain; *ω*_*e*_ is the angular velocity of the motor; *L*_*d*_, *L*_*q*_ is the *d-q*-axis components of the inductor; *ψ*_*f*_ represents the permanent magnet chain.

Since the armature winding inductance *L*_*d*_ = *L*_*q*_ in this model, the torque and motion equations can be written as

$$T_{e}=\frac{3}{2}p_{n}i_{q}\left[i_{q}\left(L_{q}-L_{d}\right)+\psi_{f}\right]$$ (4)

$$J{\dot{\omega }}_{m}=T_{e}-T_{L}-B\omega_{m}$$ (5)

Where: *T*_*e*_ is the electromagnetic torque; *P* is the number of motor pole pairs; *J* is the rotational inertia; *ω*_m_ is the mechanical angular speed of the motor; *T*_*L*_ is the load torque; *B* is the friction coefficient.

In a speed control system, the externally applied load torque *T*_*L*_ is usually considered a disturbance. Then the above equation can be changed to

$${\dot{\omega }}_{m}=\frac{3p\psi_{f}}{2J}i_{q}-\frac{T_{L}}{J}-\frac{B}{J}\omega_{m}$$ (6)

Several simple parameter transformation equations are involved in the model design.


$$\left\{\begin{array}{l} \omega_{e}=n_{p}\omega_{m}\\ N_{r}=\frac{30}{\pi }\omega_{m}\\ \theta_{e}=\int \omega_{e}dt \end{array}\right.$$ (7)

Where: *N*_*r*_ is the speed of the motor, *r* / min.

## Design and analysis of the controller

### Control principle of SMC and FTSMC

Sliding-mode variable-structure control systems first appeared in the 1960s and were proposed by Utkin [32], but due to hardware limitations at that time, the switching frequency could not meet the requirements of the controller. Later, after years of development, sliding mode variable structure control has become an important part of modern control science.

The sliding mode controller works weakly related to the control object parameters and disturbances and is relying on the conversion term in it to adjust the control quantity, which is only related to the error of the control quantity concerning the reference value, so this feature makes the controller robust and responsive. The application of sliding mode controllers in doubly-fed induction generators has thus become the focus of many scholars. In the literature [33], ordinary sliding mode control was used in the machine-side and grid-side control of doubly-fed induction generators to cope with the shocks brought by voltage dips to the generators, and the results showed that sliding mode control can effectively suppress DC voltage overcharge during low voltage ride-through of doubly-fed induction generators. The literature [34] applied fractional-order sliding mode to the machine side converter control of a doubly-fed induction generator and used an adaptive algorithm to adjust the controller parameters; the literature [35] applied higher-order terminal sliding mode control to the network side converter control of a doubly-fed induction generator, which not only eliminated the error of the control object to zero in a limited time but also effectively suppressed the jitter.

Sliding mode control is a control strategy for variable structure control systems. The fundamental difference between this control strategy and conventional control is the discontinuity of control, i.e. a switching characteristic that makes the system structure change over time. This characteristic allows the system to move up and down under certain conditions along a defined state trajectory with small subsets and high frequency, which is called "sliding mode". This sliding mode can be designed and is independent of the system parameters and perturbations. Therefore, the system in the sliding mode has good robustness.

However, there is also a drawback of the slide-mode controller, because the state trajectory of the system does not slide along the slide-mode surface when it runs to the slide-mode surface, but goes up and down, which is also called the jitter phenomenon of the slide-mode controller. In order to reduce the impact of the jitter phenomenon, domestic and foreign scholars also try to reduce the jitter through various aspects of the design of the slide-mode variable structure controller.

The fast terminal sliding mode control can make the system state converge to zero in a limited time, which is a breakthrough from the normal sliding mode control in the linear sliding mode surface condition of the state asymptotic convergence, and the dynamic performance of the system is better than the normal sliding mode control. And compared with the linear sliding mode control, the terminal sliding mode control has no switching term, which can effectively eliminate jitter. Fast terminal sliding mode control brings a new development direction for sliding mode control theory. However, both methods have the problem of control singularity, so research and discussion of non-singular fast terminal sliding mode control are needed to solve the problems.

### Design of non-singular fast terminal sliding mode controller

Take the state variable of the PMSM system as

$$\left\{\begin{array}{l} x_{1}=\omega_{ref}-\omega \\ x_{2}={\dot{x}}_{1}=-\dot{\omega } \end{array}\right.$$ (8)

Where: *ω*_*ref*_ is the given speed; *ω* is the actual speed

Combining Eq (5) yields

$$\left\{\begin{array}{l} \frac{\text{d}x_{1}}{\text{d}t}=-\frac{\text{d}\omega }{\text{d}t}=-\frac{p}{J}\left(\frac{3}{2}p\psi_{f}i_{q}-T_{L}\right)\\ \frac{\text{d}x_{2}}{\text{d}t}=-\frac{{\text{d}}^{2}\omega }{\text{d}t^{2}}=-\frac{3p^{2}}{2J}\psi_{f}\frac{\text{d}i_{q}}{\text{d}t} \end{array}\right.$$ (9)

Let $D=\frac{3p^{2}}{2J}\psi_{f},\;U=\frac{\text{d}i_{q}}{\text{d}t}$,and the system state space can be obtained

$$\left[\begin{array}{l} {\dot{x}}_{1}\\ {\dot{x}}_{2} \end{array}\right]=\left[\begin{array}{cc} 0 & 1\\ 0 & 0 \end{array}\right]\left[\begin{array}{l} x_{1}\\ x_{2} \end{array}\right]+\left[\begin{array}{c} 0\\ -D \end{array}\right]U$$ (10)

The non-singular fast terminal slip surface proposed in this paper is given by the following equation.


$$s=x_{2}+cx_{1}+\frac{1}{\beta }x_{1}^{\frac{p_{0}}{q_{0}}}$$ (11)

In the formula: *c*∈*R*^+^, *β*∈*R*^+^, *p*_0_, *q*_0_ ∈ *N* is an odd number,and 1 < *p*_0_ / *q*_0_ < 2.

In order to suppress the sliding mode jitter problem and to ensure non-singularity, a convergence law is designed using a convergence approach with terminal attractors.


$$\dot{S}=-\phi s-\gamma S^{\frac{m}{n}}$$ (12)

In the formula: *c*∈*R*^+^, *β*∈*R*^+^, *p*_0_, *q*_0_ ∈ *N* is an odd number,and 0 < *m* / *n* < 1.

Choosing Eq (11) for the system slip surface, combining it with Eq (10) and taking the partial derivative of *s* yields

$$\dot{s}=\left(c+\frac{p_{0}}{\beta q_{0}}x_{1}^{\frac{p_{0}}{q_{0}}-1}\right)x_{2}-D\frac{\text{d}i_{q}}{\text{d}t}$$ (13)

The PMSM fast non-singular terminal sliding mode controller is then derived from Eqs (11) and (13) as follows.


$$i_{q}=\frac{1}{D}\int \left[\left(\phi s+\gamma s^{\frac{m}{n}}\right)+\left(c+\frac{p_{0}}{\beta q_{0}}x_{1}^{\frac{p0}{q0}-1}\right)x_{2}\right]\text{d}t$$ (14)

### Controller stability analysis

According to the second theory of Lyapunov stability, the Lyapunov function is chosen as $V_{v}=\frac{1}{2}s^{2}$, then.


$${\dot{V}}_{v}=s\dot{s}$$ (15)

Substituting Eq (11) and Eq (13) into Eq (15) yields

$$\begin{array}{c} {\dot{V}}_{v}=s\dot{s}=s\left[\left(c+\frac{p_{0}}{\beta q_{0}}x_{1}^{\frac{p0}{q0}-1}\right)-D\frac{\text{d}i_{q}}{\text{d}t}\right]\\ =\left(x_{2}+cx_{1}+\frac{1}{\beta }x_{1}^{\frac{p0}{q0}}\right)\left[\left(c+\frac{p_{0}}{\beta q_{0}}x_{1}^{\frac{p0}{q0}-1}\right)-D\frac{\text{d}i_{q}}{\text{d}t}\right] \end{array}$$ (16)

where *c, β* all belong to R^+^,1 < *p*_0_ / *q*_0_ < 2, *φ*∈*R*^+^,*γ*∈*R*^+^, *m*, *n*∈*N*

so

$${\dot{V}}_{v}=\left(x_{2}+cx_{1}+\frac{1}{\beta }x_{1}^{\frac{p0}{q0}}\right)\left[\left(c+\frac{p_{0}}{\beta q_{0}}x_{1}^{\frac{p0}{q0}-1}\right)-D\frac{\text{d}i_{q}}{\text{d}t}\right]\leq 0$$ (17)

According to Lyapunov second stability theory:

If there exists a function with continuous partial derivatives, satisfying the following conditions.

1. *V*(*t*) ≥ 0;
2. *V*(*t*) ≤ 0;

After the above theoretical proof, it is shown that the designed speed controller satisfies the stability theory. It is proved that the speed controller can make the system error converge quickly.

### Deviation coupling control strategy based on position information feedback

**Fig 2:**
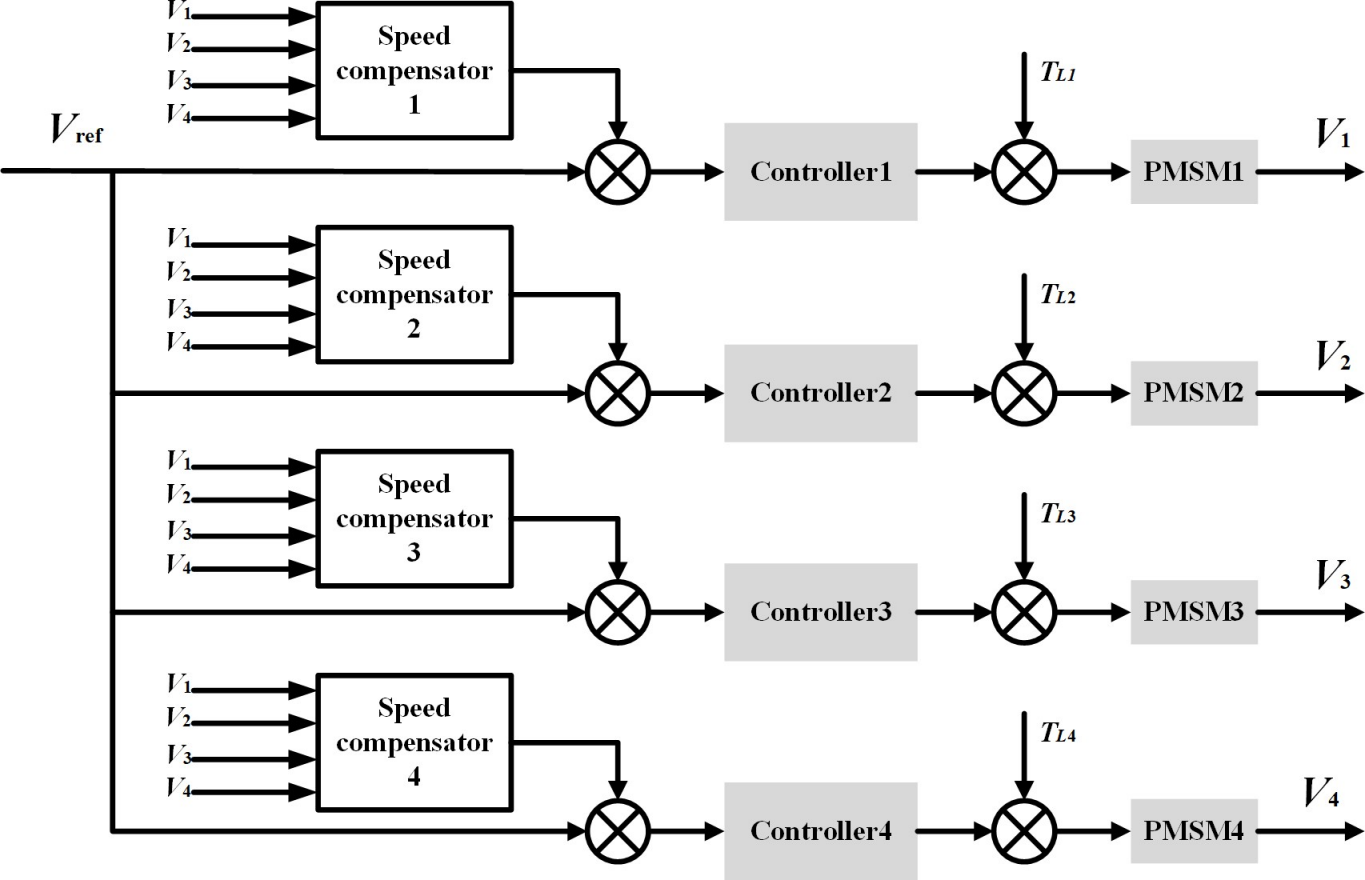
Structure diagram of multi-motor deviation coupling control strategy.

**Fig 3:**
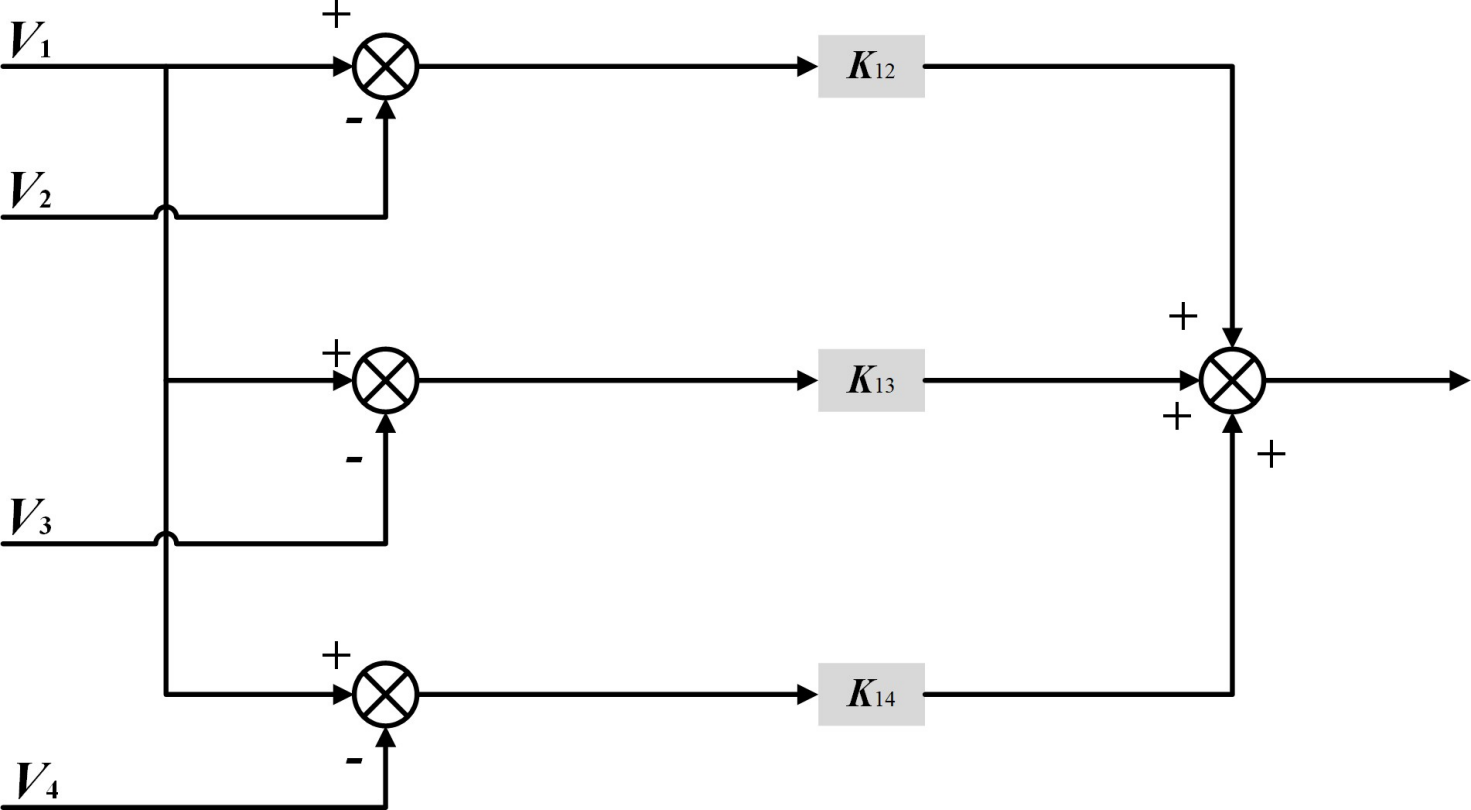
Classical deviation coupling speed compensator structure diagram.

The deviation coupling control is improved from the cross-coupling control in the multi-motor scenario, and the compensation signal is jointly determined by the operating state change of each motor in the system. Its control structure is shown in Fig 2, and the motor speed compensator structure is shown in Fig 3. The actual speed of the controlled motor and the actual speed of each other motor are differenced and multiplied by an appropriate coefficient *k*_*ij*_, which is summed up as the control input compensation of the controlled motor, and the coefficient is usually taken as the ratio of the rotational inertia J of each motor, i.e, *k*_*ij*_ = *k*_*i*_/*k*_*j*_.

**Fig 4:**
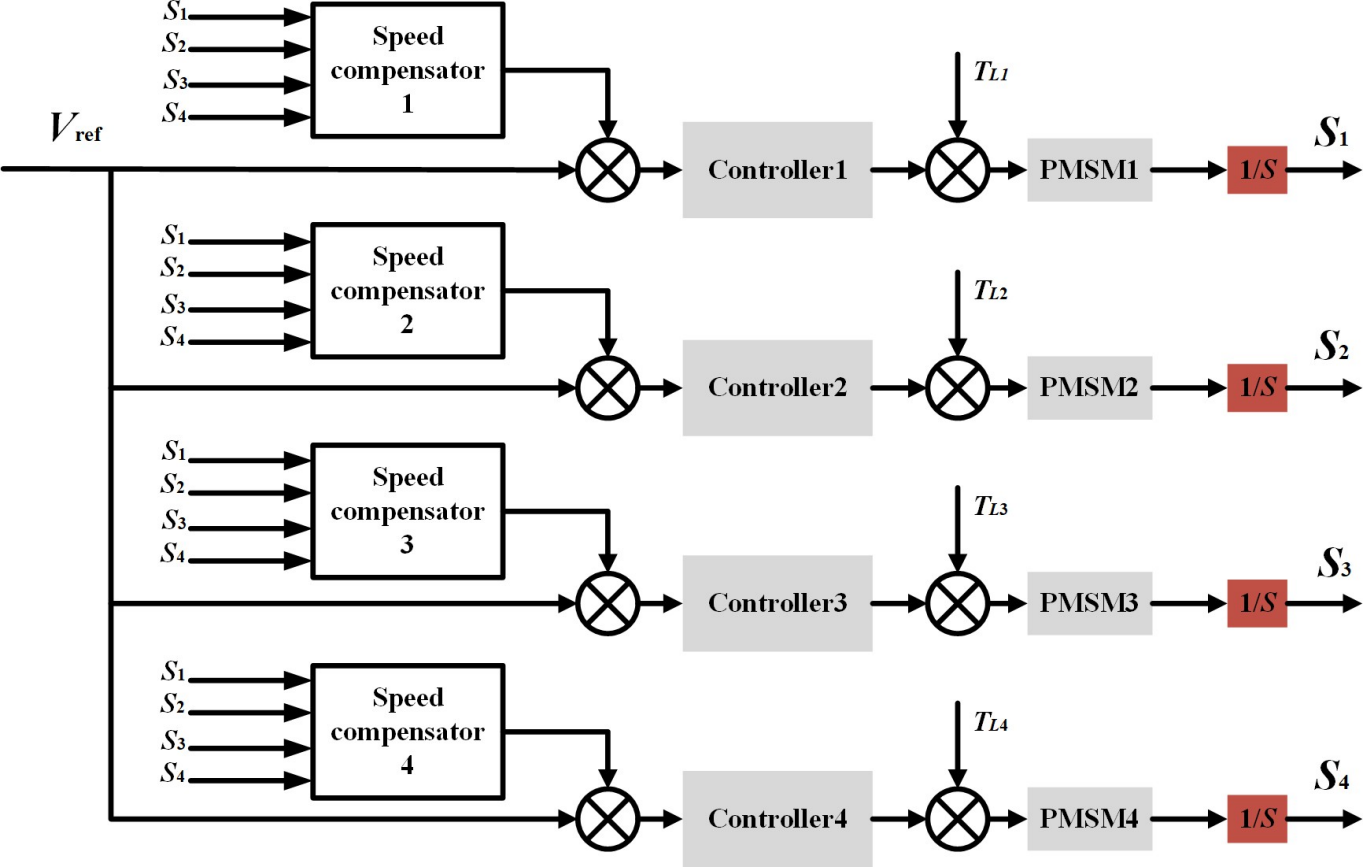
Structure diagram of multi-motor deviation coupling control strategy based on position information feedback.

**Fig 5:**
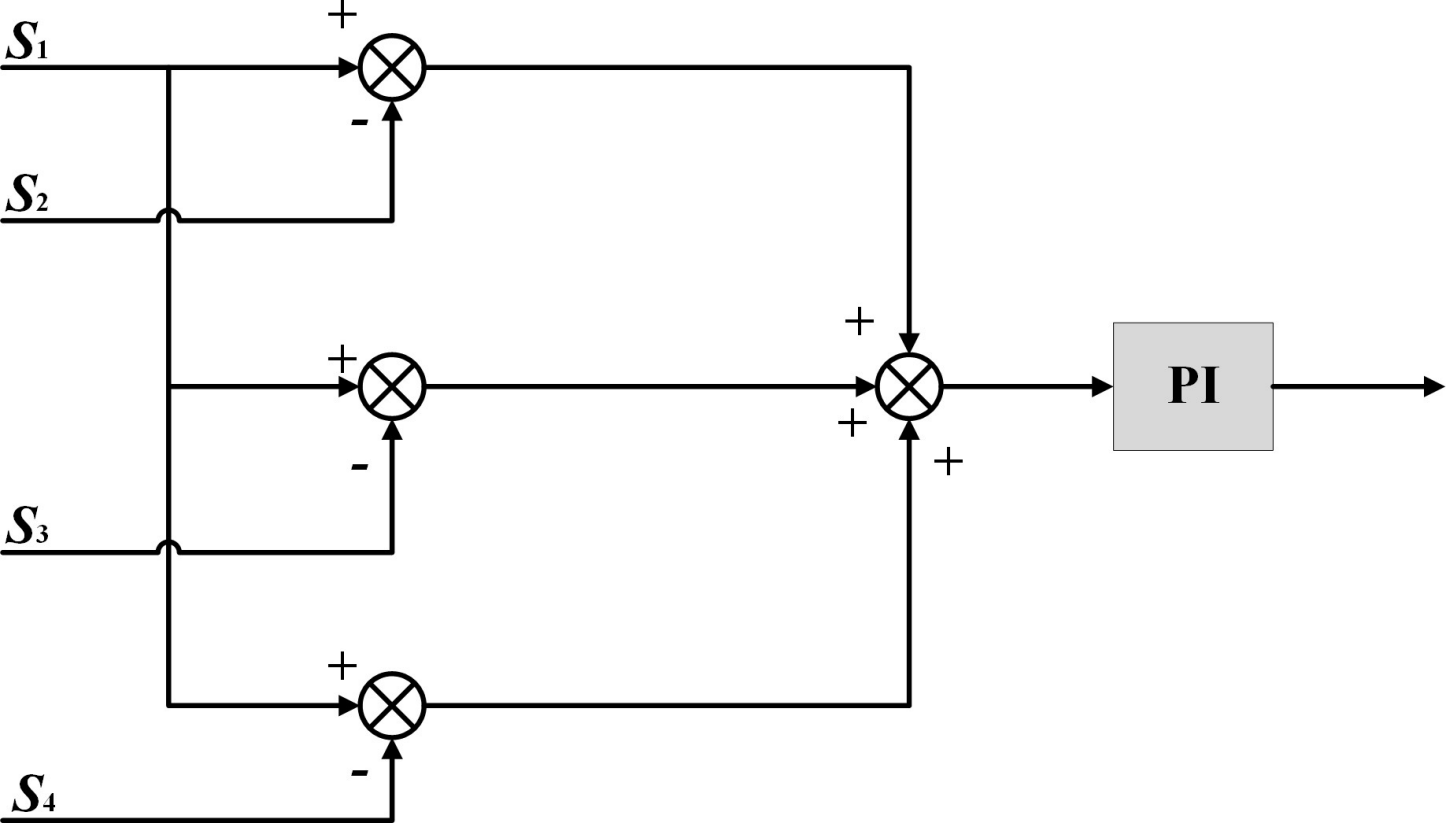
Structure diagram of improved deviation coupling position compensator.

**Fig 6:**
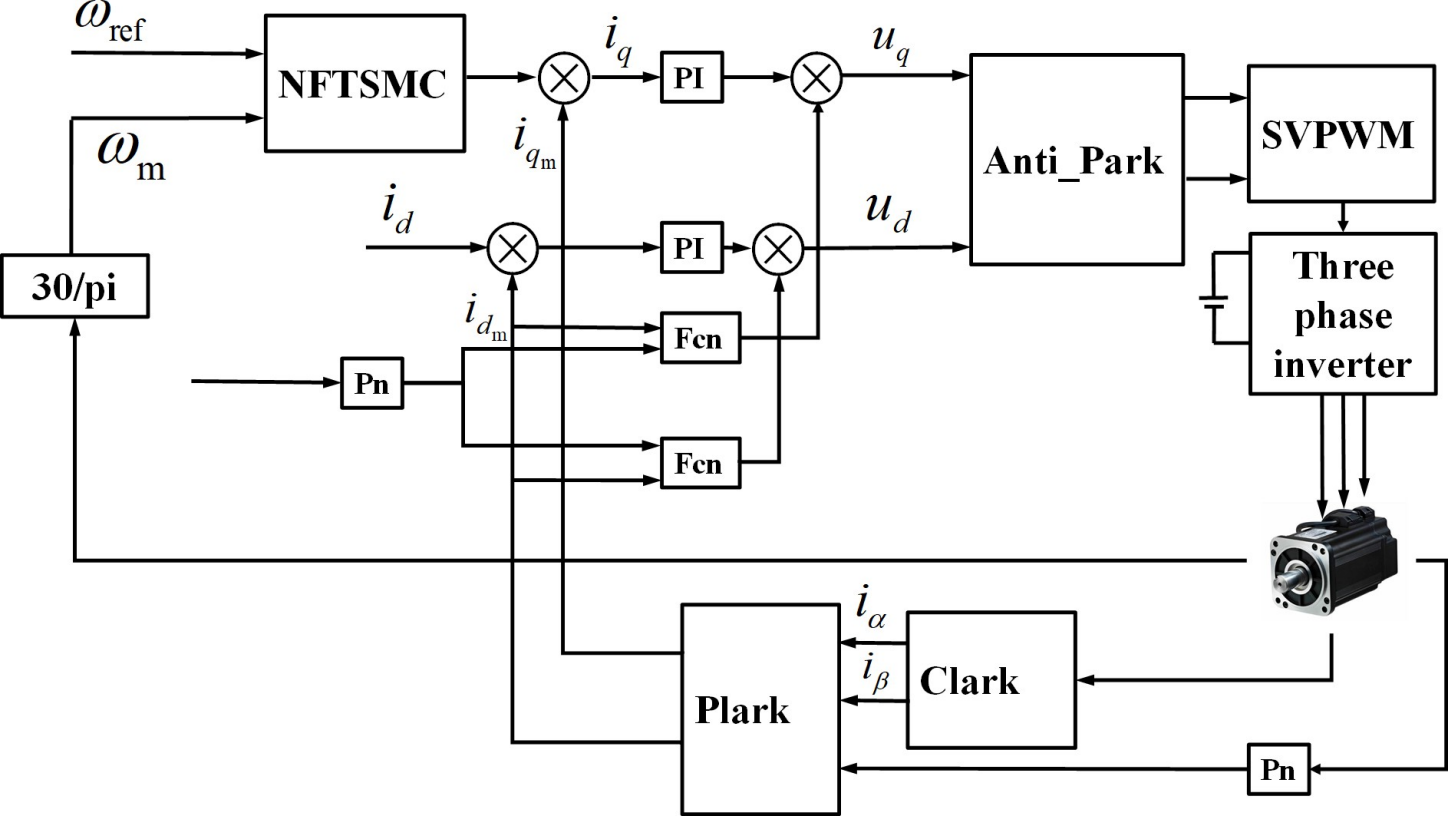
Single motor control structure block diagram.

Since this article is a high-precision control of the position of multiple motors, the deviation coupling control strategy based on position information feedback is obtained based on the general structure of Fig 2 for speed integration and feedback, as shown in Fig 4. And this paper adopts a PI controller instead of the fixed initial gain to achieve feedforward control of the motor. Its structure is shown in Fig 5. Once the interference appears, before the controlled quantity changes, the PI regulator produces the control effect, that is, directly based on the detected speed of other motors according to a particular law to quickly eliminate the following error between motors, so that it is stable convergence to zero, thus ensuring that the system has excellent synchronization performance at the same time so that the system obtains better dynamic and static performance. One of the single motor control structure block diagrams is shown in Fig 6.

## Simulation

### Simulation parameter setting

With the permanent magnet synchronous motor as the primary control object, the parameters of the motor model in the d-q rotation coordinate system are set in Table 2, the debugging parameters in the SMC controller are specified in Table 3, the debugging parameters in the FTSMC controller are set in Table 4, the debugging parameters in the NFTSMC controller are set in Table 5, and the debugging parameters in the modified deviation coupling control structure are *k*_*p*_ = 5 and *k*_*i*_ = 1.

**Table 2:** Motor parameter settings.

Parameters	Value
*R*_*s*_/Ω	2.875
*L*_*s*_ / *mH*	0.0085
*ψ_f_* / *W*b	0.175
*J* / (*kg*•*m*^2^)	0.003
*P*	4
*B* / (*N*•*m*•*s*)	0.008

**Table 3:** SMC parameter settings.

Parameters	Value
*c*	60
*Mu*	200
*q*	300

**Table 4:** FTSMC parameter settings.

Parameters	Value
*K*_*1*_	diag(8,12,6,7,5)
*K*_*2*_	diag(9,8,10,6,7)
*K*_*3*_	diag(13,12,8,10,9)
*c*_*1*_	15
*c*_*2*_	12
*q*	5
*h*	13
*p*	3
*g*	7

Note: diag(*) is the diagonal matrix

**Table 5:** NFTSMC controller parameter settings.

Parameters	Value
*p*_*0*_	0.9
*q*_*0*_	0.00025
*m*	75
*n*	55
*ϕ*	51
*β*	27
*c*	400000
*r*	1000000

### Simulation comparison test of multi-motor under different control algorithms

In order to verify that the NFTSMC control cited in this paper has better position synchronization performance than SMC control and FTSMC control, this simulation is based on MATLAB/Simulink to build an experimental platform for simulation.

Δ*s*_*ij* max_ is the maximum synchronization error between the ith motor and the jth motor in r. The larger its value is, the worse the synchronization performance of the system is. The maximum synchronization error between each motor is calculated according to the position error variation curve of each motor of the above three control structures.

#### Working condition setting

**Fig 7:**
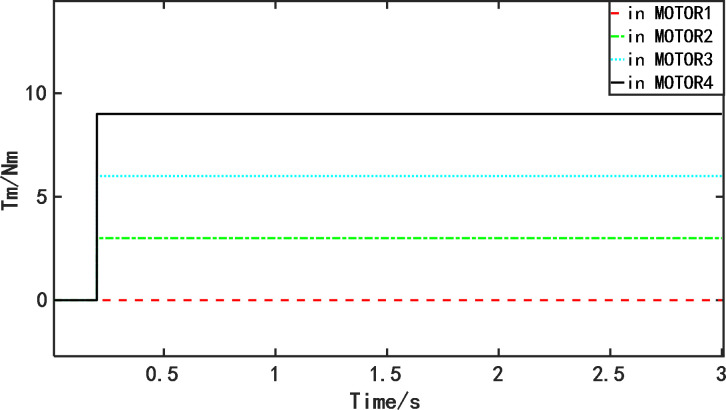
Variation of the sudden addition of disturbance torque for four motors.

Three groups of multi-motor position synchronous control systems are designed, each group has four PMSMs, and all three groups of multi-motor position synchronous control systems use IDCC as the multi-motor position synchronous control structure, and NFTSMC, FTSMC and SMC are used as the controllers of the three groups of multi-motor control systems for simulation and comparison tests respectively. The tracking target speed is set to 1000r/min, the overall simulation time is 3s, and the sudden load torque of the four motors is 0Nm, 3Nm, 6Nm and 9Nm at t = 0.2s. The simulation is based on MATLAB/Simulink, so the sudden load torque is set up as a step module in the Tm interface of the permanent magnet synchronous motor model. The load torque variation is shown in Fig 7. The specific debugging parameters of the simulated controlled motor are shown in Table 2, the debugging parameters of the SMC controller are shown in Table 3, the debugging parameters of the FTSMC controller are shown in Table 4, the debugging parameters of the NFTSMC controller are shown in Table 5, and the IDCC position compensator parameters *k*_*p*_ = 5 and *k*_*i*_ = 1.

#### Comparative analysis of simulation results

**Fig 8:**
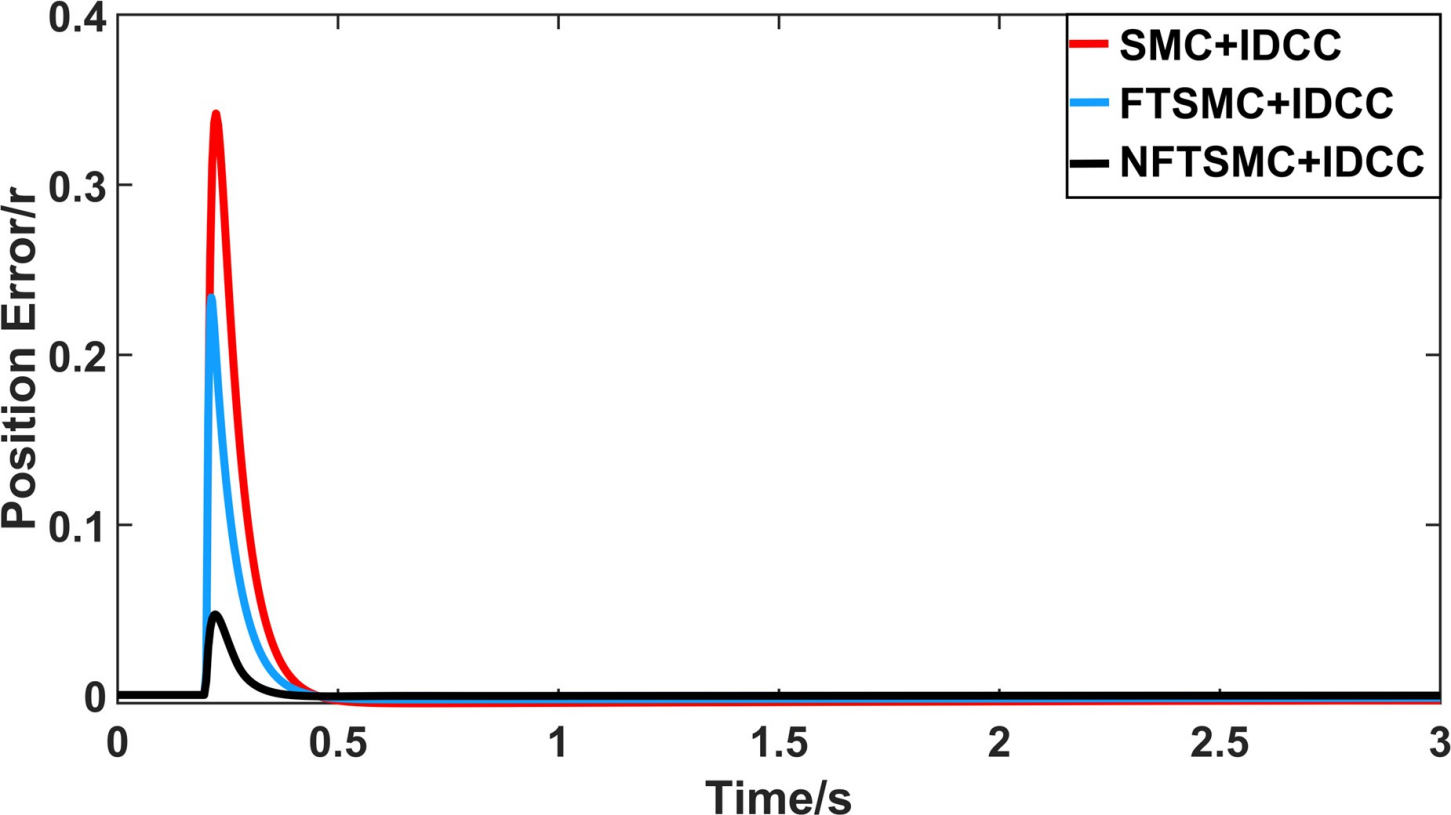
Position error between motor 1 and motor 2 for different algorithms.

**Fig 9:**
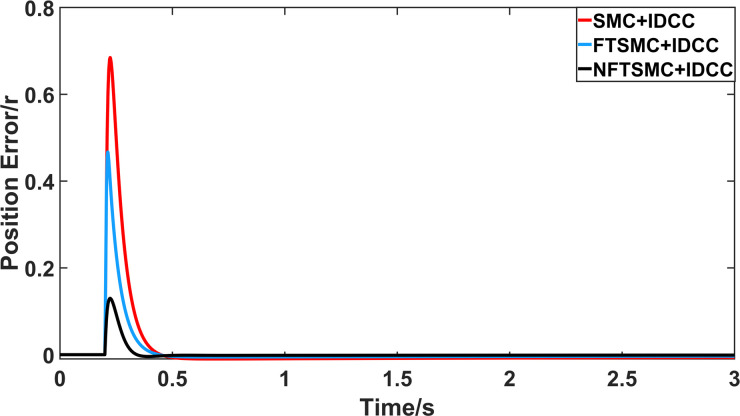
Position error between motor 1 and motor 3 for different algorithms.

**Fig 10:**
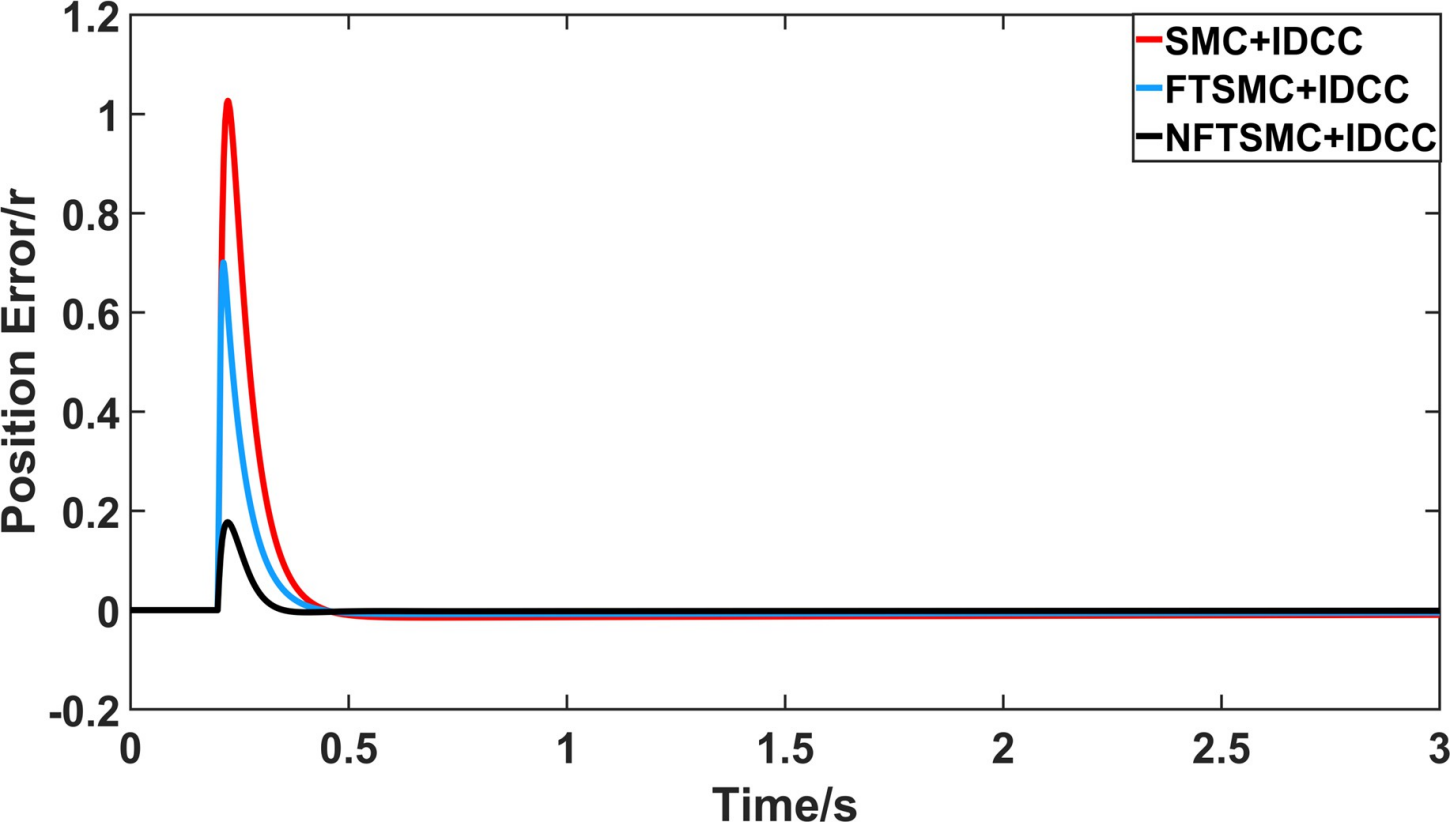
Position error between motor 1 and motor 4 for different algorithms.

**Fig 11:**
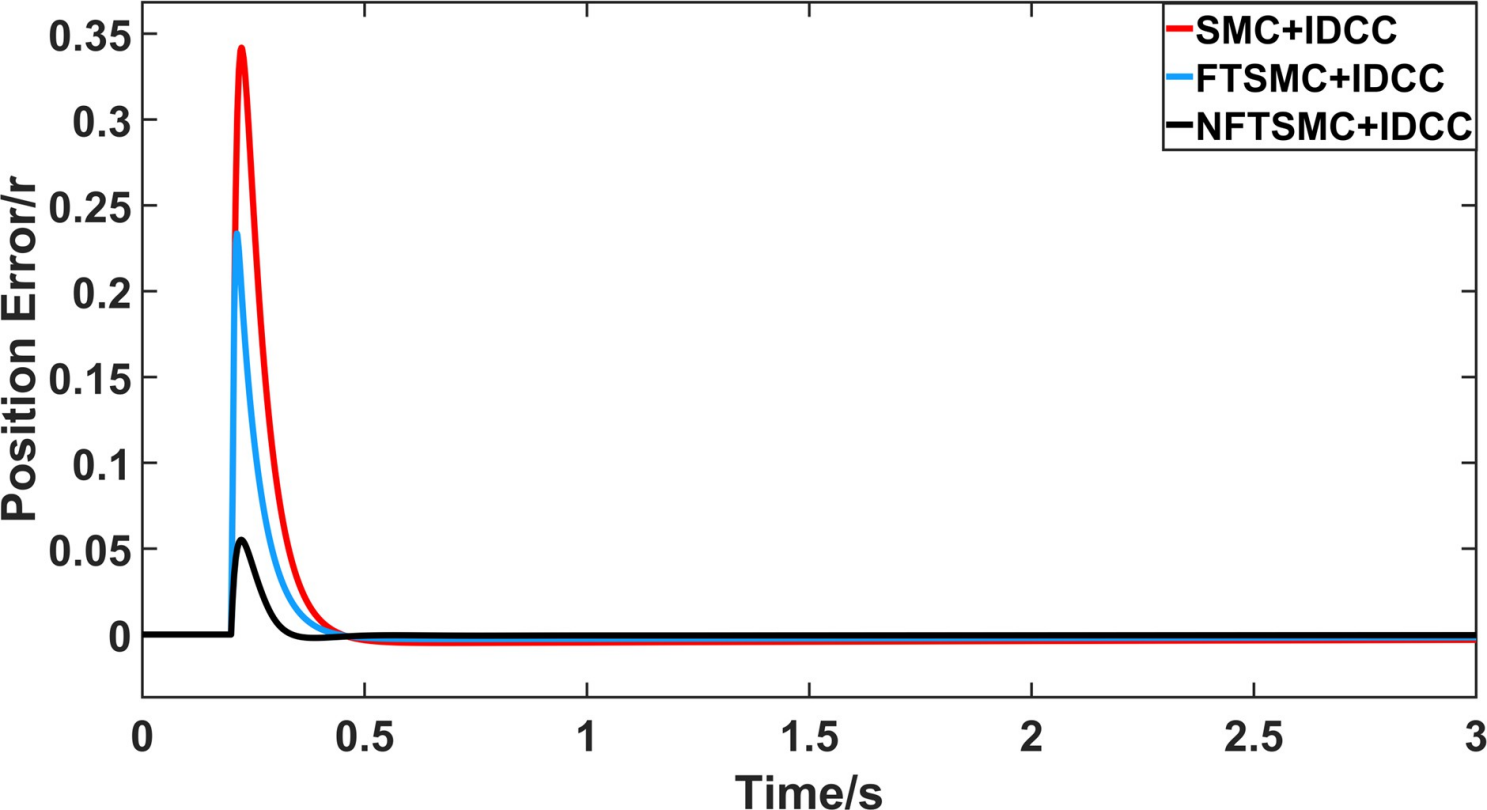
Position error between motor 2 and motor 3 for different algorithms.

**Fig 12:**
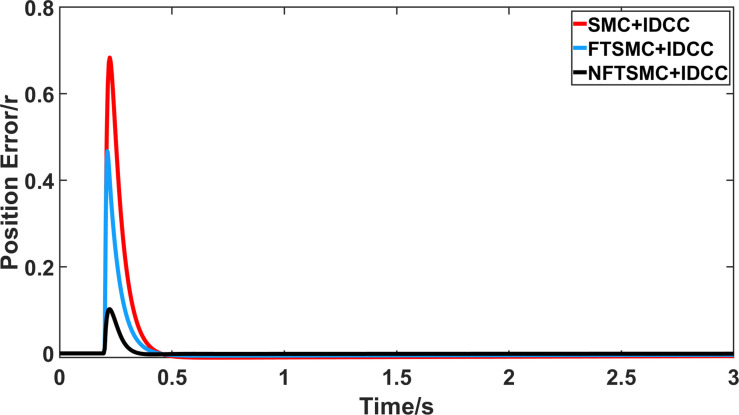
Position error between motor 2 and motor 4 for different algorithms.

**Fig 13:**
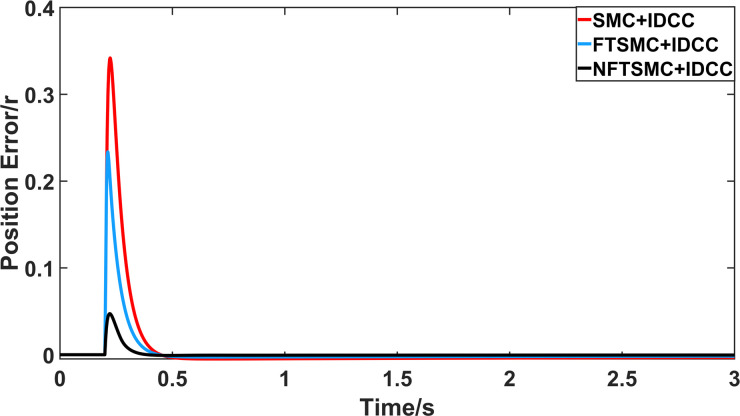
Position error between motor 3 and motor 4 for different algorithms.

From Figs 8–13 and Table 6, it can be seen that the multi-motor position synchronization control system based on NFTSMC control is better than the other two algorithms, both in terms of the maximum position error value and the position error adjustment time, where the position error under SMC control is the largest compared to the other two algorithms. This paper analyzes the total error by adding the position error data between the six pairs of motors compared with the four pairs of motors in Figs 8–13 and Table 6 and making them two by two. Therefore, the total position error of the SMC-controlled multi-motor position synchronous control system is 3.39r, and the absolute position error of the FTSMC-controlled multi-motor position synchronous control system is 2.325r, while the total position error of the NFTSMC-controlled multi-motor position synchronous control system is only 0.553. FTSMC by 2.837r and 1.772r improves the multi-motor coupling by 83.68% and 76.22%, respectively. Specifically, since the difference in sudden load between Motor1 and Motor4 is the largest, its position error is also the largest. The maximum error in the position between Motor1 and Motor4 in its multi-motor system under SMC control reaches 1.01r, while the multi-motor position synchronization control system based on NFTSMC control is only 0.174r, which significantly reduces the displacement error. This dramatically reduces the displacement error and shortens the error adjustment time. Finally, it is concluded that the improved deviation-coupled multi-motor synchronous control based on NFTSMC control, the performance of the coupling between motors on multi-motor synchronous control all reflect better synchronization and appropriate control method, and the superiority of the cooperation of the control structure.

**Table 6:** Maximum synchronization error between motors under three control algorithms.

Controller	Δ_12max_	Δ_13max_	Δ_14max_	Δ_23max_	Δ_24max_	Δ_34max_
SMC	0.34	0.68	1.01	0.34	0.68	0.34
FTSMC	0.23	0.46	0.7	0.23	0.47	0.235
NFTSMC	0.042	0.127	0.174	0.054	0.09	0.046

### Simulation comparison test of multiple motors at different target speeds

In order to verify the multi-motor position synchronization performance of the proposed multi-motor position synchronization control system based on the combination of non-singular fast terminal sliding mode control and improved deviation coupling, this simulation also uses a permanent magnet synchronous motor as the object to be controlled in this simulation. The specific parameters of the control motor are shown in Table 2, the commissioning parameters of the SMC controller are shown in Table 3, the commissioning parameters of the FTSMC controller are shown in Table 4, the commissioning parameters of the NFTSMC controller are shown in Table 5, and the parameters of the improved deviation coupling position compensator *k*_*p*_ = 5 and *k*_*i*_ = 1.

#### Working condition setting

Three groups of multi-motor position synchronous control systems are designed, four PMSMs are created in each group of multi-motor systems, and NFTSMC is used as the controller. The RCC, DCC, and IDCC are used as the synchronous control structures of the three groups of multi-motor position synchronous control systems for simulation and comparison tests. The above three groups of multi-motor position synchronous control systems are simulated and compared three times; the first time set the target speed to 800r/min; the first time, set the target speed to 1000r/min; the third time, set the target speed to 1500r/min, the load torque is 0, the simulation time is 3s, at 0.2s, the four PMSM load torque is disturbed, the first motor load The load torque of the first motor is unchanged and runs typically; the additional torque of the second motor is 3Nm at t = 0.2s; the extra torque of the third motor is 6Nm at t = 0.2s; the additional torque of the fourth motor is 9Nm at t = 0.2s.

#### Comparative analysis of simulation results

**Fig 14:**
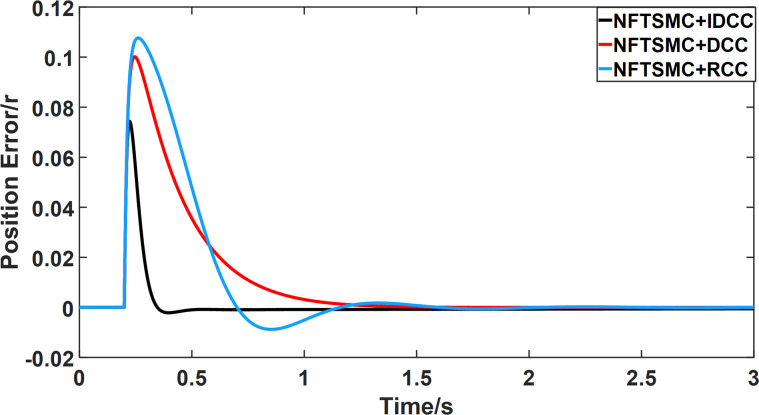
Position error between motor 1 and motor 2 for different multi-motor control structures at 800 r/min.

**Fig 15:**
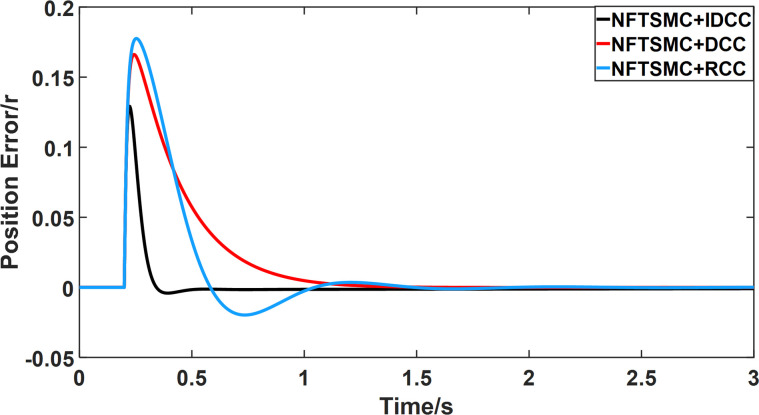
Position error between motor 1 and motor 3 for different multi-motor control structures at 800 r/min.

**Fig 16:**
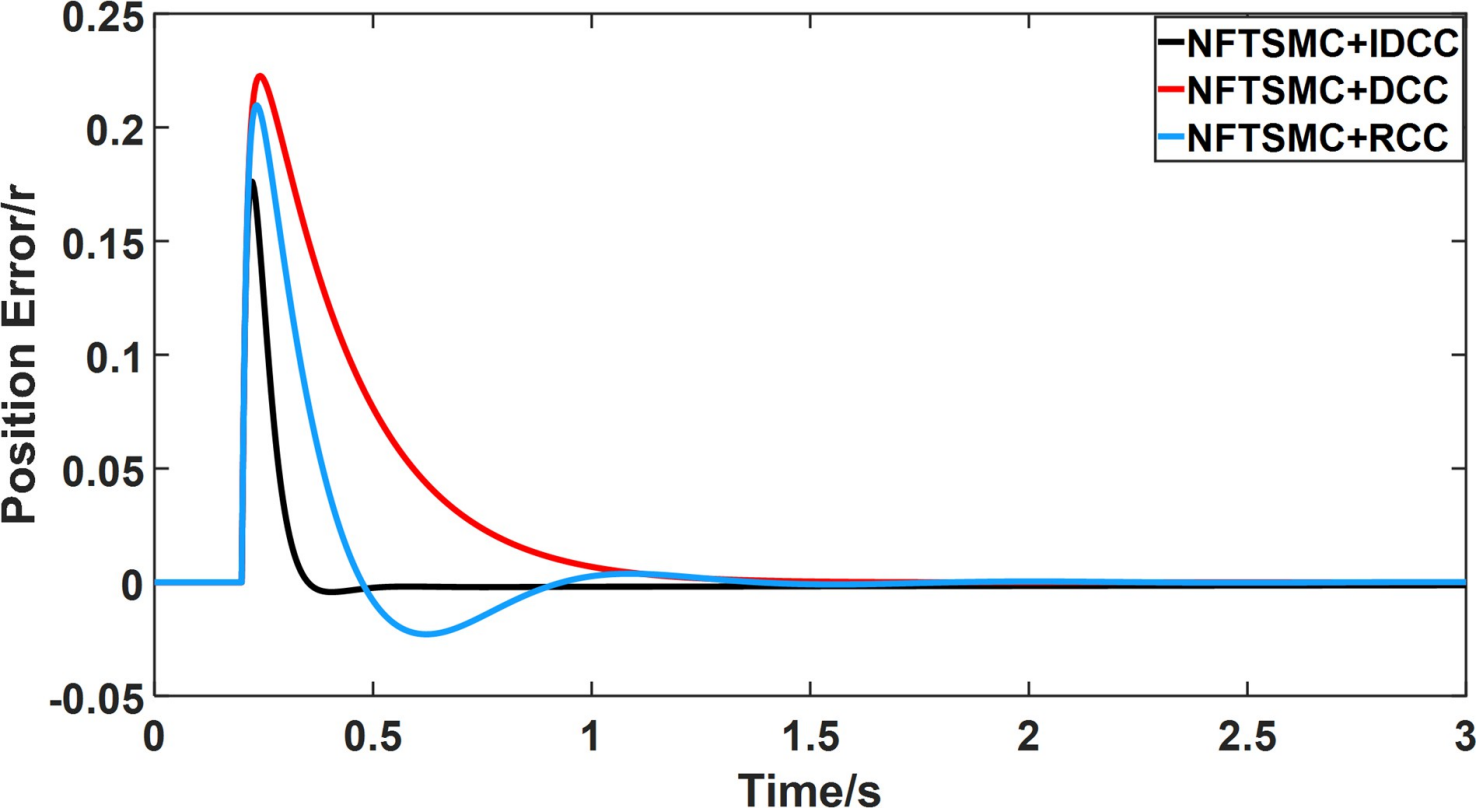
Position error between motor 1 and motor 4 for different multi-motor control structures at 800 r/min.

**Fig 17:**
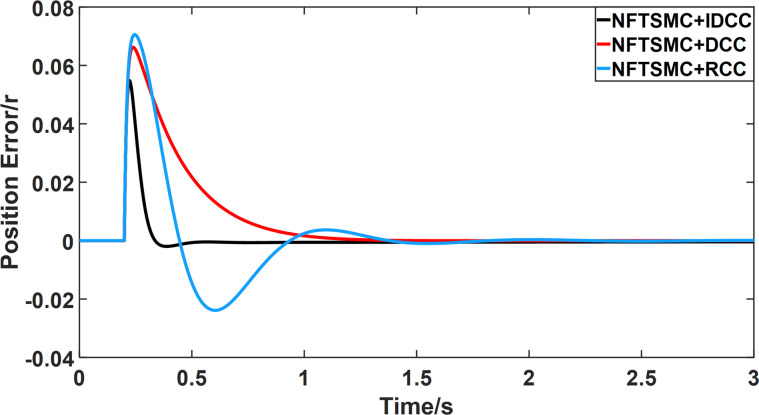
Position error between motor 2 and motor 3 for different multi-motor control structures at 800 r/min.

**Fig 18:**
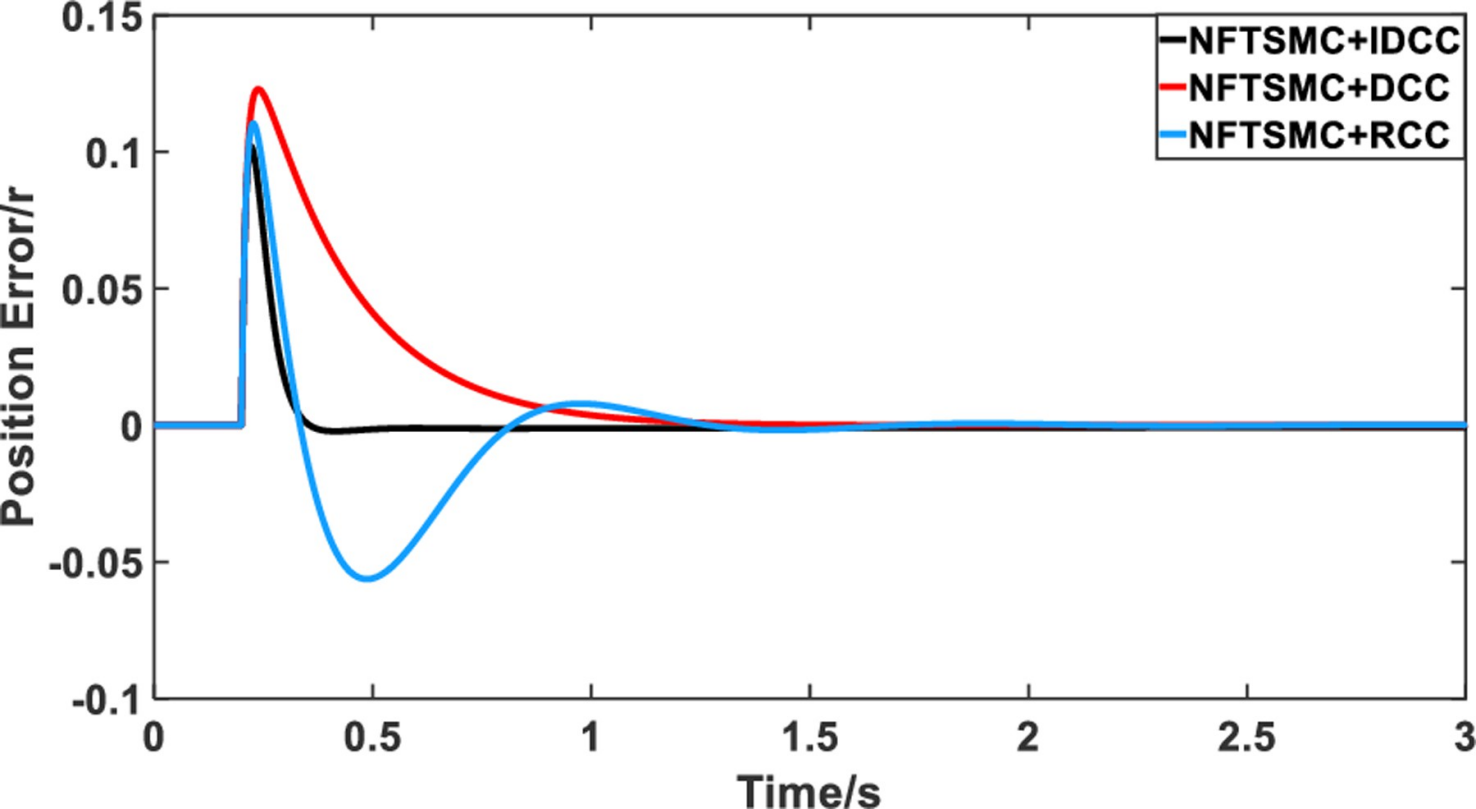
Position error between motor 2 and motor 4 for different multi-motor control structures at 800 r/min.

**Fig 19:**
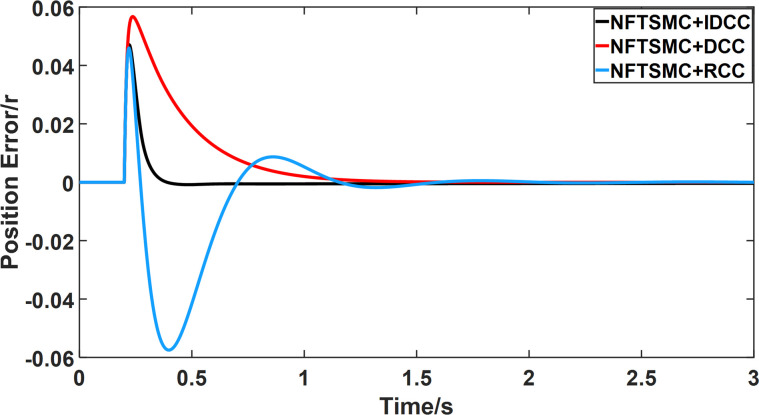
Position error between motor 3 and motor 4 for different multi-motor control structures at 800 r/min.

**Fig 20:**
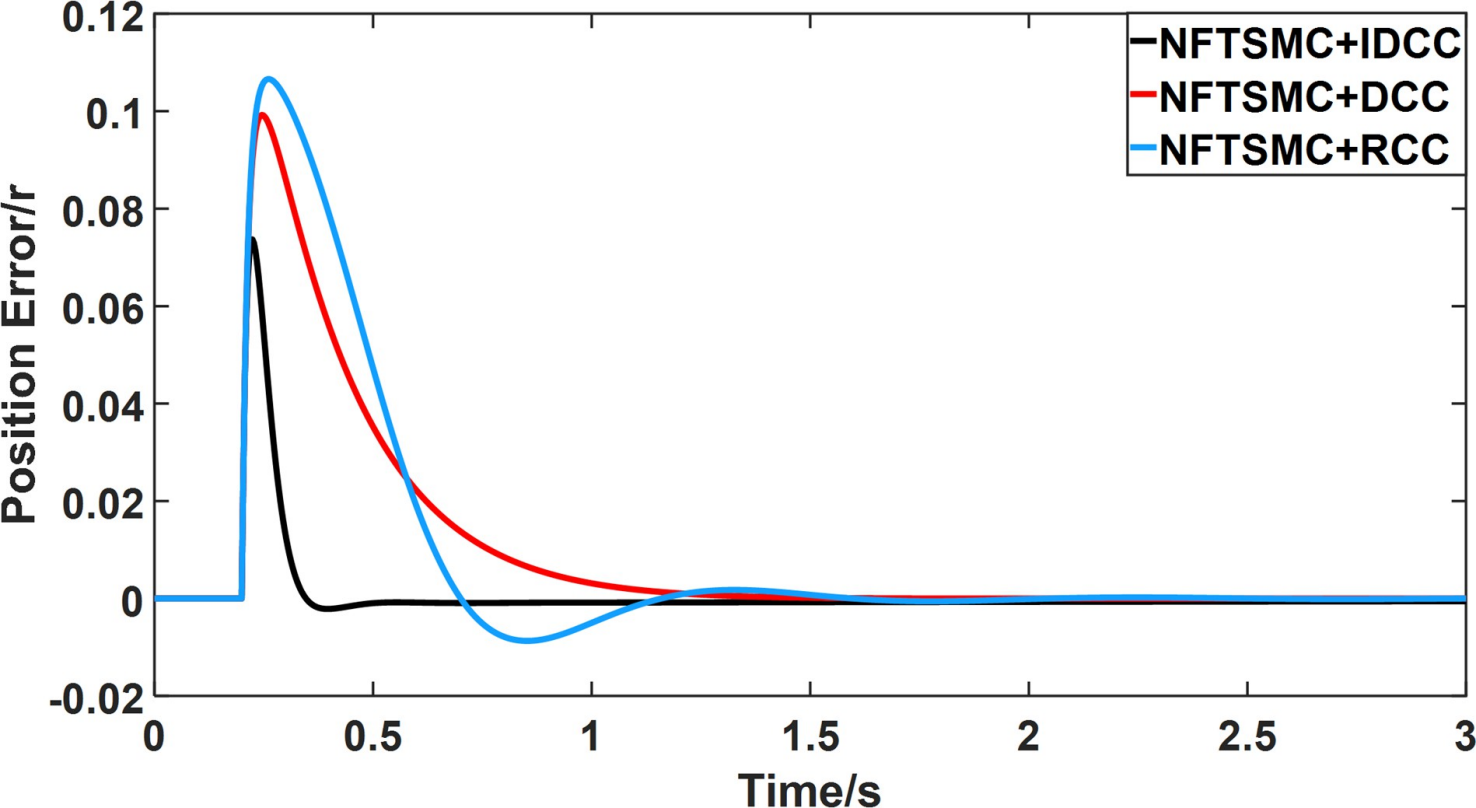
Position error between motor 1 and motor 2 for different multi-motor control structures at 1000 r/min.

**Fig 21:**
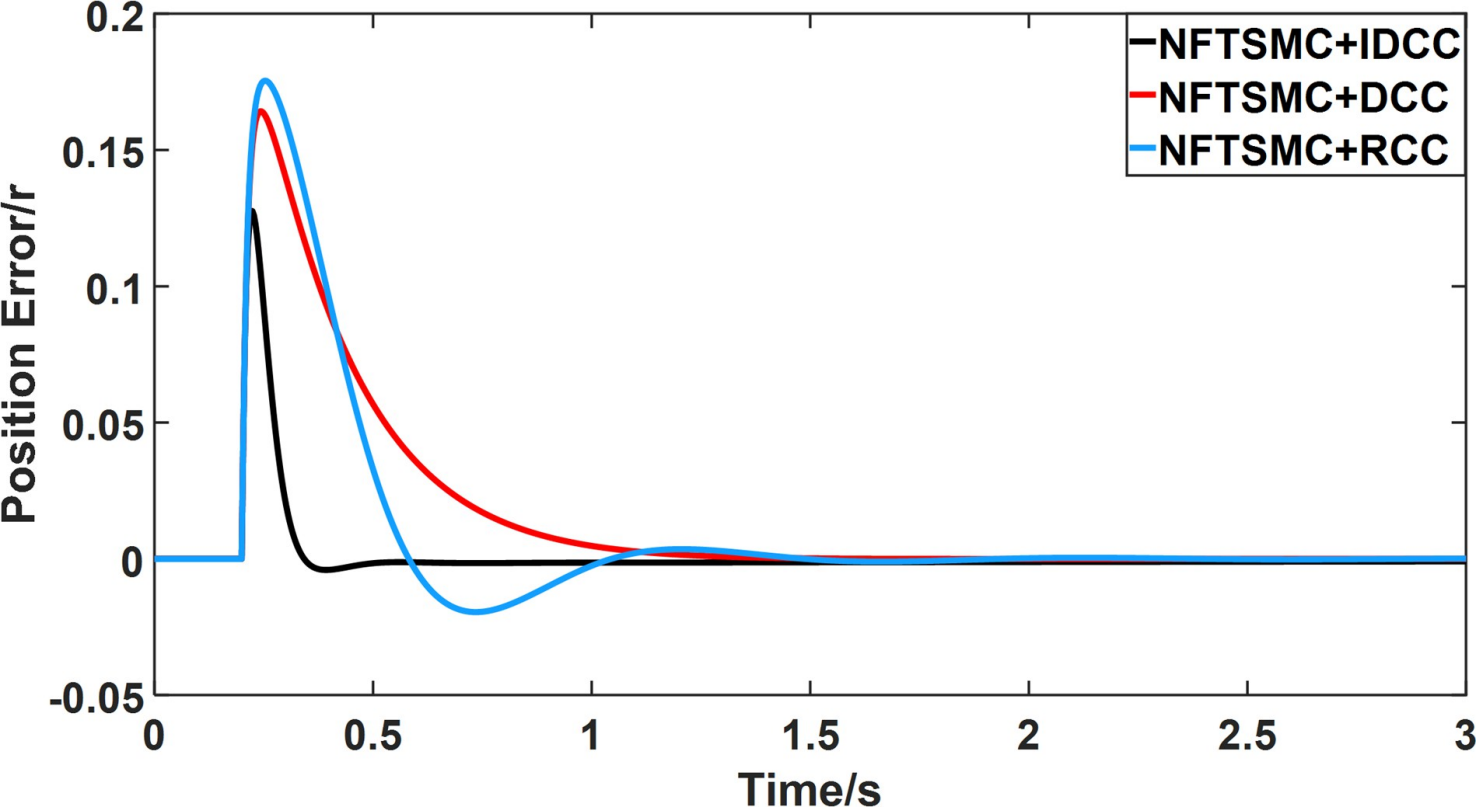
Position error between motor 1 and motor 3 for different multi-motor control structures at 1000 r/min.

**Fig 22:**
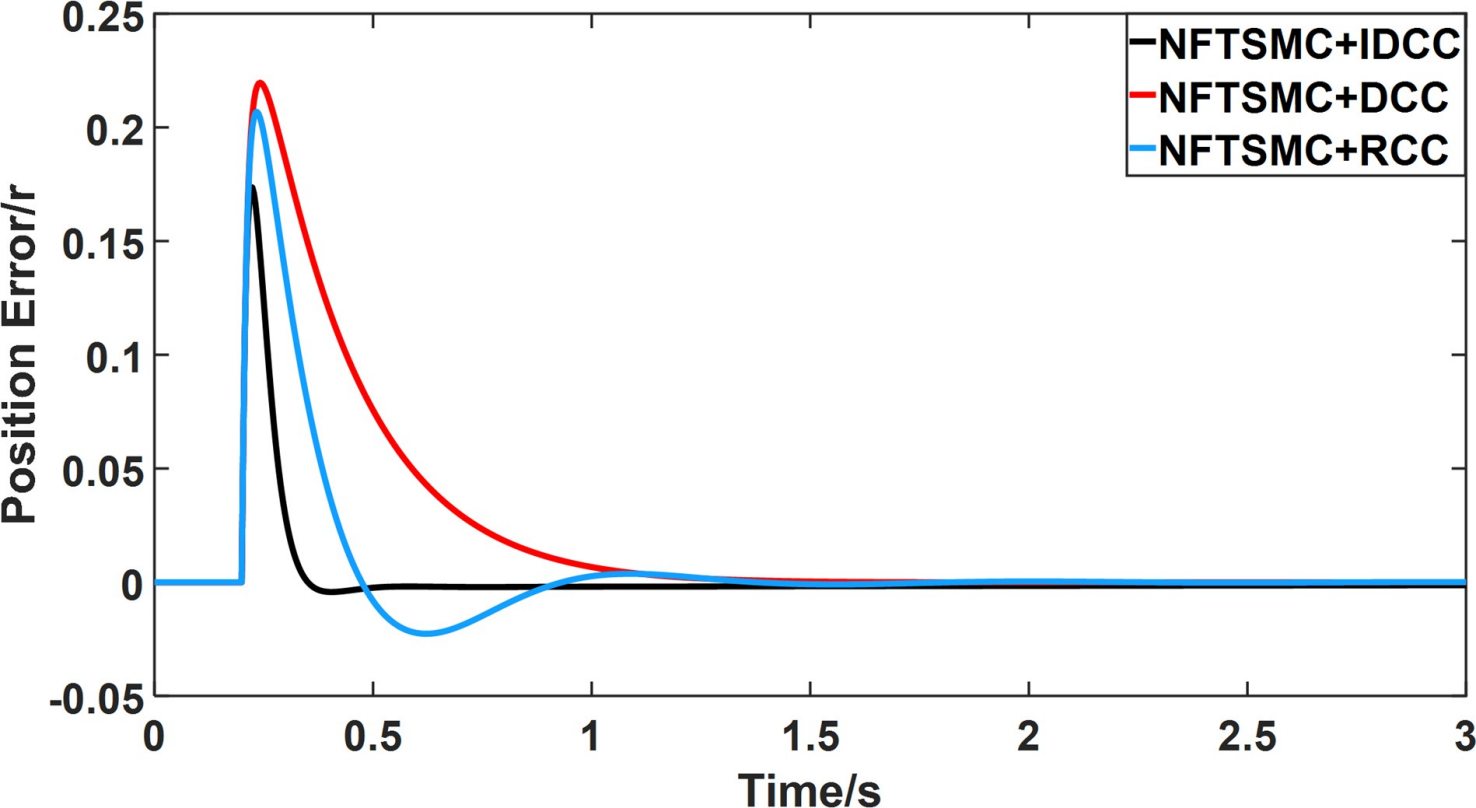
Position error between motor 1 and motor 4 for different multi-motor control structures at 1000 r/min.

**Fig 23:**
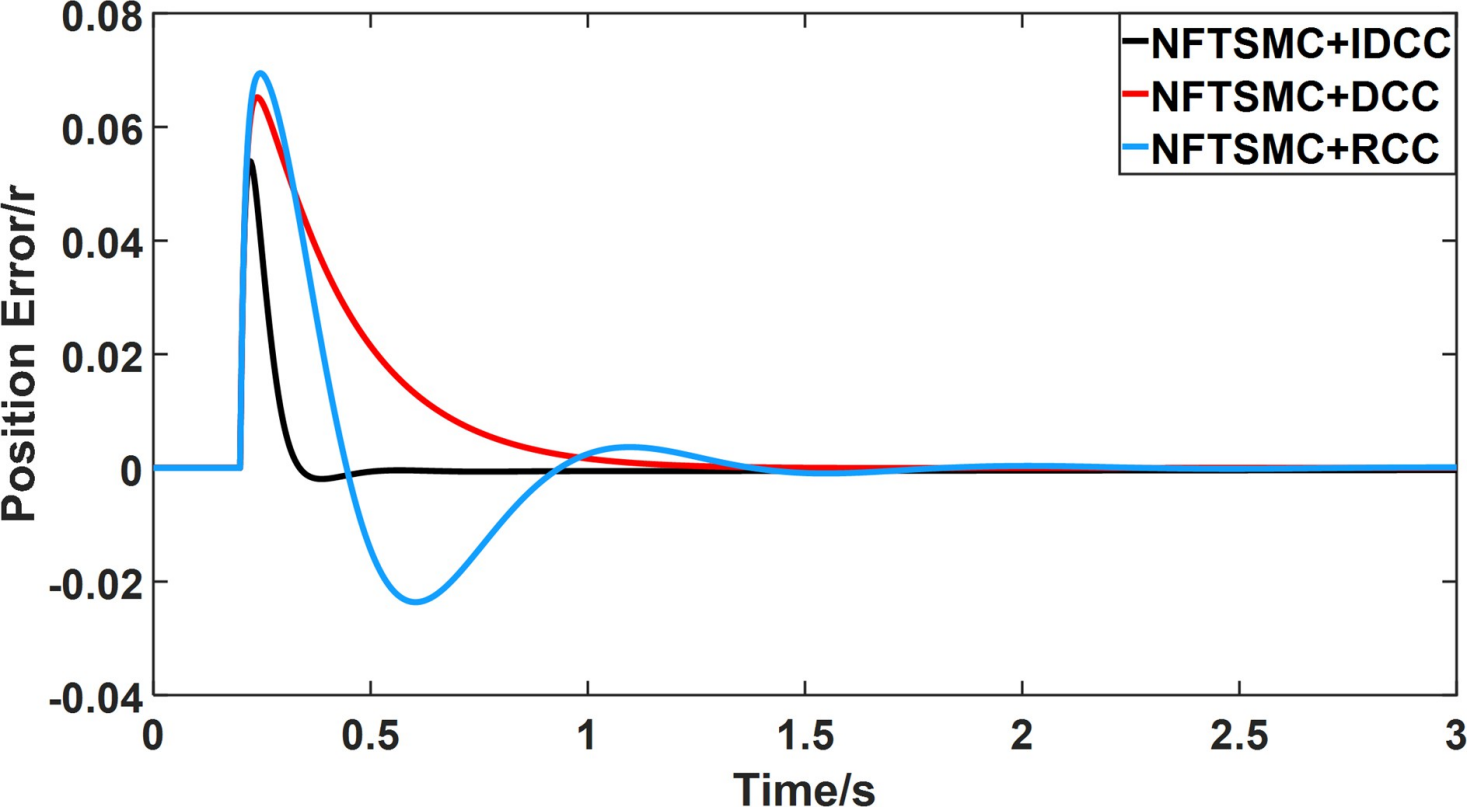
Position error between motor 2 and motor 3 for different multi-motor control structures at 1000 r/min.

**Fig 24:**
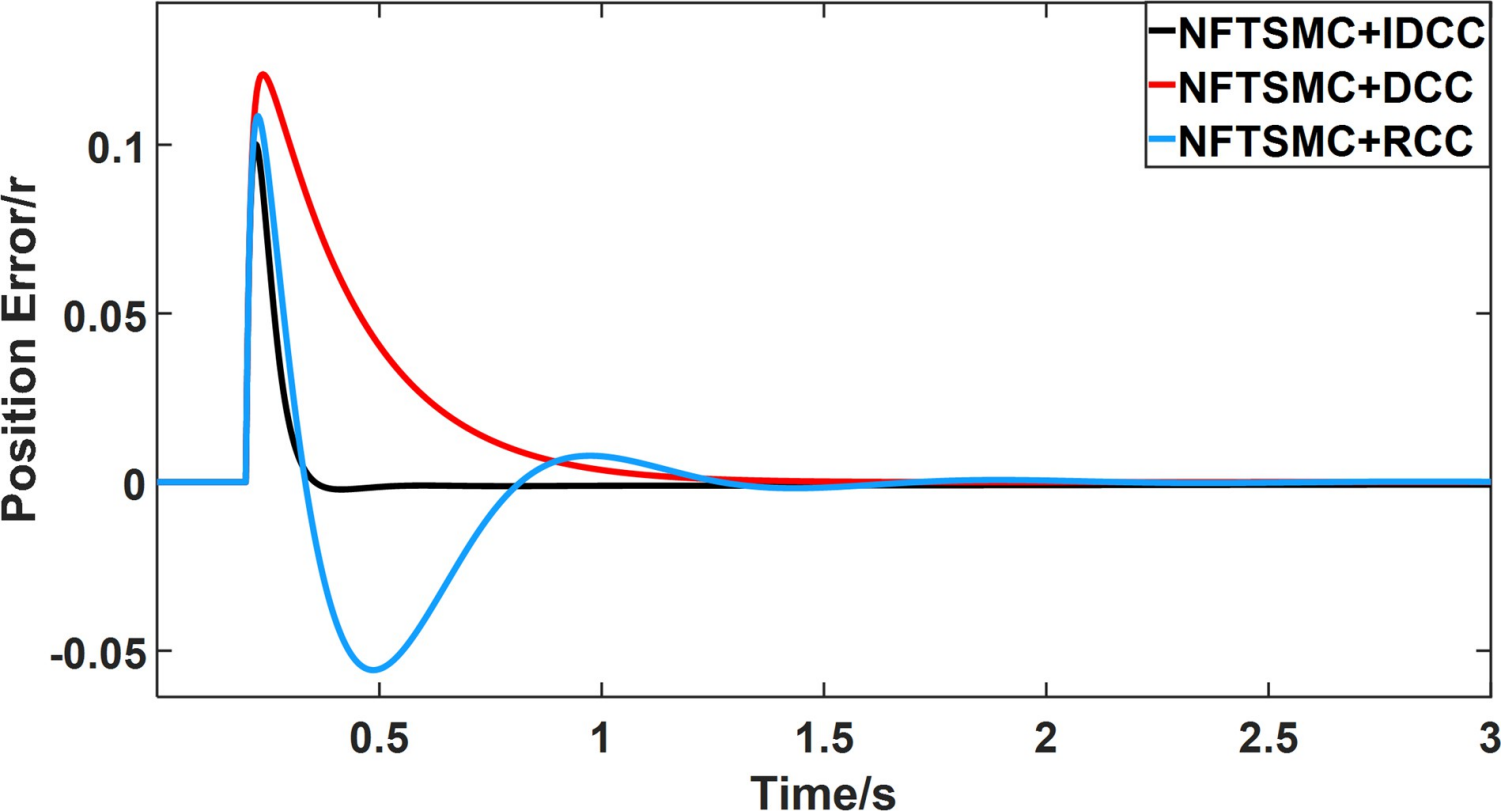
Position error between motor 2 and motor 4 for different multi-motor control structures at 1000 r/min.

**Fig 25:**
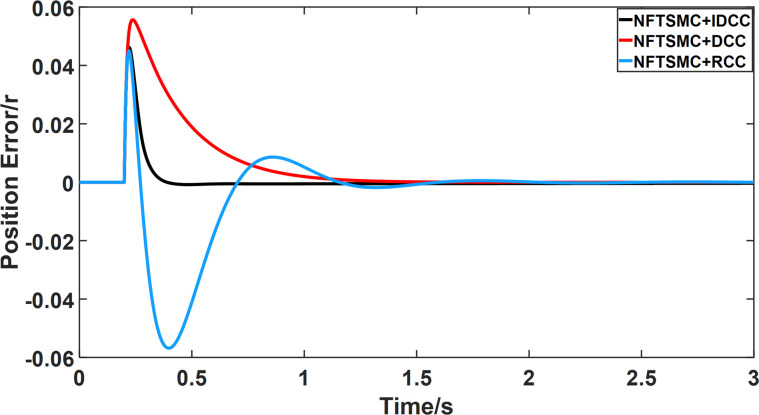
Position error between motor 3 and motor 4 for different multi-motor control structures at 1000 r/min.

**Fig 26:**
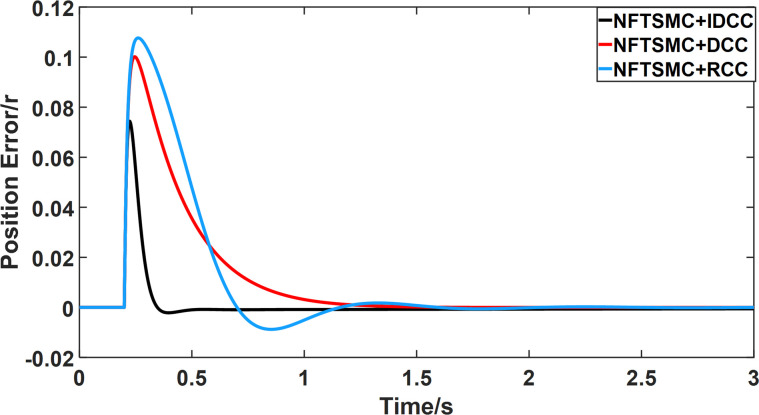
Position error between motor 1 and motor 2 for different multi-motor control structures at 1500 r/min.

**Fig 27:**
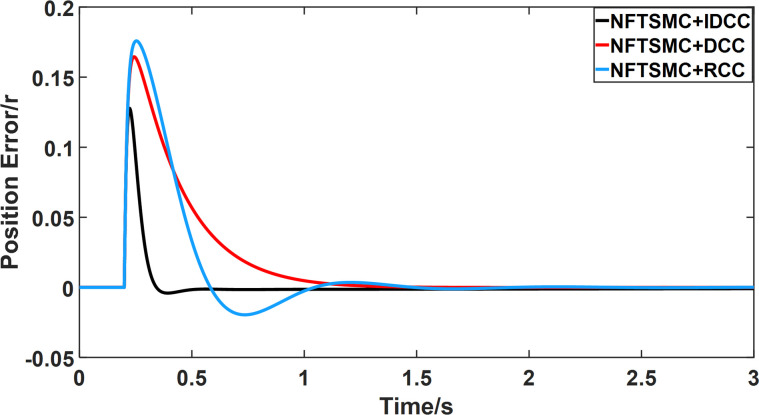
Position error between motor 1 and motor 3 for different multi-motor control structures at 1500 r/min.

**Fig 28:**
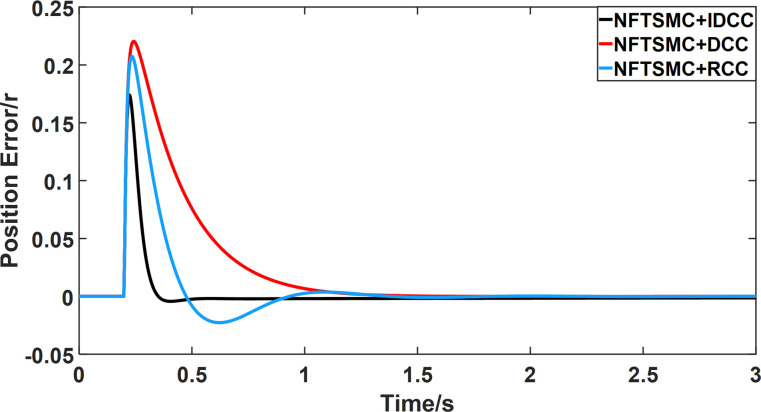
Position error between motor 1 and motor 4 for different multi-motor control structures at 1500 r/min.

**Fig 29:**
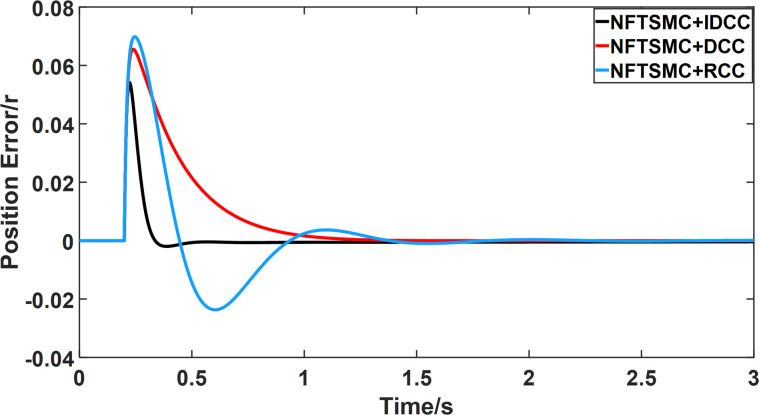
Position error between motor 2 and motor 3 for different multi-motor control structures at 1500 r/min.

**Fig 30:**
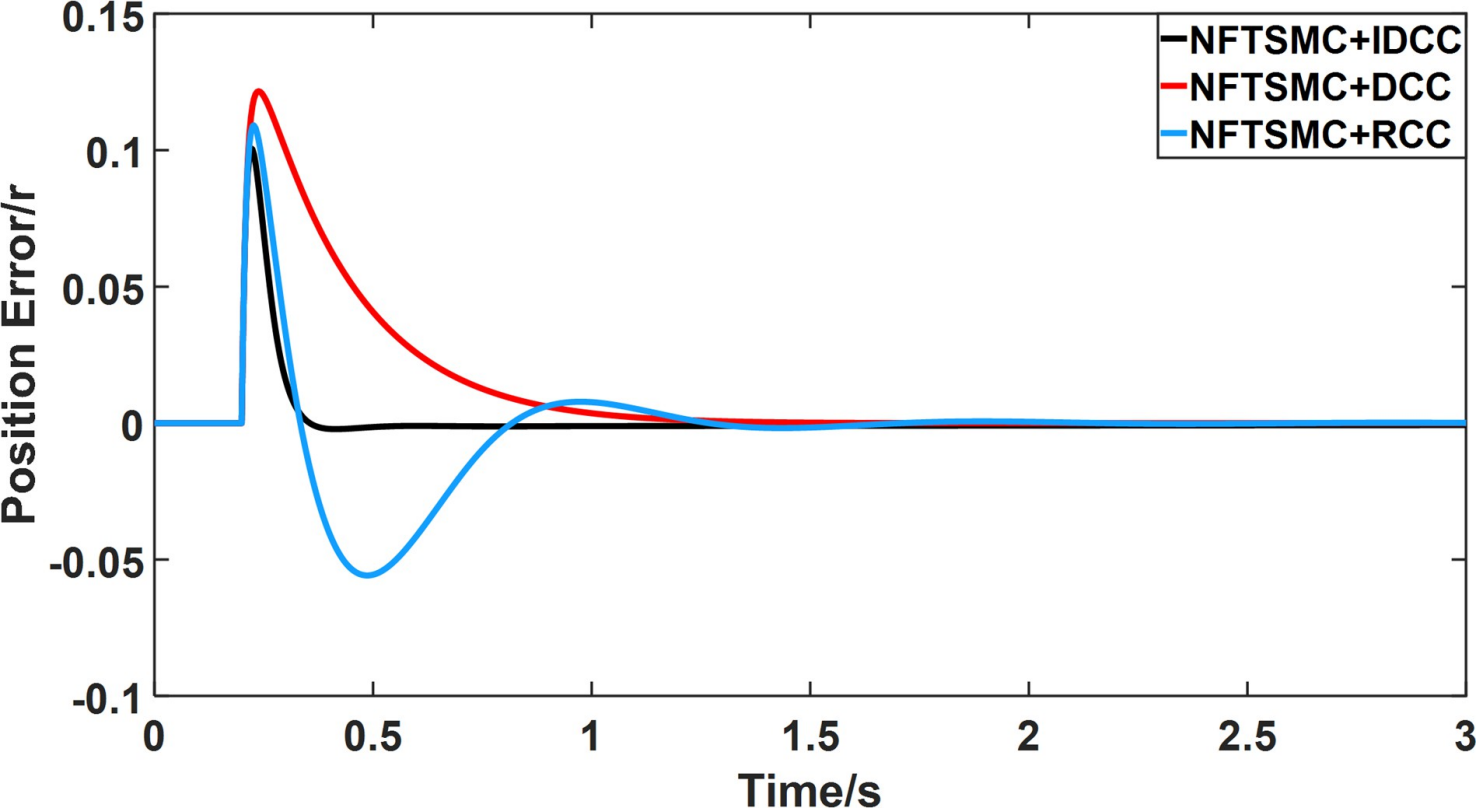
Position error between motor 2 and motor 4 for different multi-motor control structures at 1500 r/min.

**Fig 31:**
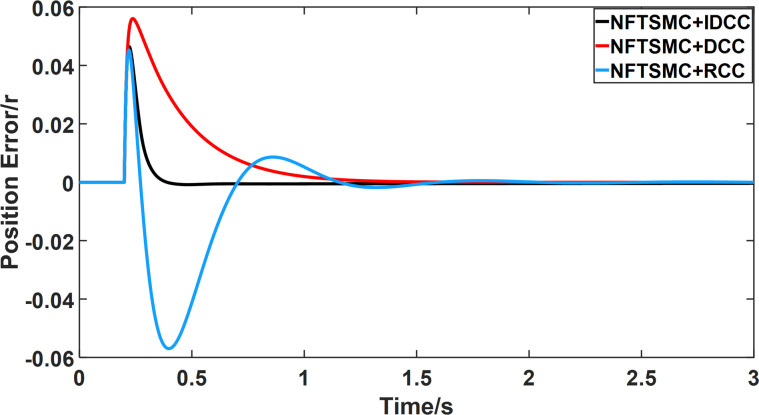
Position error between motor 3 and motor 4 for different multi-motor control structures at 1500 r/min.

From Figs 14–31, it can be seen that there is no load torque disturbance during 0–0.2s time, and all three control structures can achieve synchronous control of four PMSM positions, and at 0.2s, the load of each motor changes abruptly. At 1000r/min, the displacement synchronization error under DCC is the largest, and the maximum speed error between the first motor and the fourth motor reaches 0.221r. The total error of the same two motors under RCC control also comes a position error of 0.213r. The error under the improved deviation-coupled multi-motor synchronization control structure based on NFTSMC control is the smallest, and the maximum error under this control The maximum error under this control method is only 0.174r, which is the best control effect. The same excellent results are also achieved at 800r/min and 1500r/min speed. Overall, at 800r/min, the total error of multi-motor position under RCC is 0.743r/min and 0.73r/min under DCC, while the fundamental mistake of multi-motor position under IDCC proposed in this paper is only 0.576r/min, which is 0.167r/min and 0.154r/min less than the former two respectively. The coupling is improved by 22.47% and 21.01% respectively, showing a better synchronization performance of multi-motor position. Similarly, it can be calculated that the total error of multi-motor position under IDCC at 1000r/min and 1500r/min is the smallest compared to both RCC and DCC, which shows the universality of excellent performance. Finally, it is concluded from the simulation results that the improved deviation-coupled multi-motor synchronous control based on NFTSMC control shows better control performance at different motor operating speeds. all offer better control performance and demonstrate the superiority of the control method. The specific simulation data are shown in Tables 7–9, Once again, the improved deviation-coupled multi-motor synchronous control based on NFTSMC control shows better control performance and the superiority of the control method at different motor operating speeds.

**Table 7:** Maximum synchronization error between motors under the three control methods.

Control method	Δ_12max_	Δ_13max_	Δ_14max_	Δ_23max_	Δ_24max_	Δ_34max_
NFTSMC+RCC	0.117	0.173	0.217	0.07	0.109	0.057
NFTSMC+DCC	0.1	0.161	0.224	0.066	0.123	0.056
NFTSMC+IDCC	0.075	0.125	0.175	0.054	0.1	0.047

**Table 8:** Maximum synchronization error between motors under the three control methods.

Control method	Δ_12max_	Δ_13max_	Δ_14max_	Δ_23max_	Δ_24max_	Δ_34max_
NFTSMC+RCC	0.104	0.175	0.213	0.069	0.107	0.058
NFTSMC+DCC	0.094	0.163	0.221	0.066	0.12	0.056
NFTSMC+IDCC	0.072	0.127	0.174	0.054	0.09	0.046

**Table 9:** Maximum synchronization error between each motor under three control methods.

Control method	Δ_12max_	Δ_13max_	Δ_14max_	Δ_23max_	Δ_24max_	Δ_34max_
NFTSMC+RCC	0.106	0.175	0.209	0.07	0.109	0.058
NFTSMC+DCC	0.099	0.163	0.22	0.067	0.123	0.057
NFTSMC+IDCC	0.072	0.126	0.175	0.054	0.098	0.045

### Comparative analysis based on the simulation results of this paper and similar recent articles

**Fig 32:**
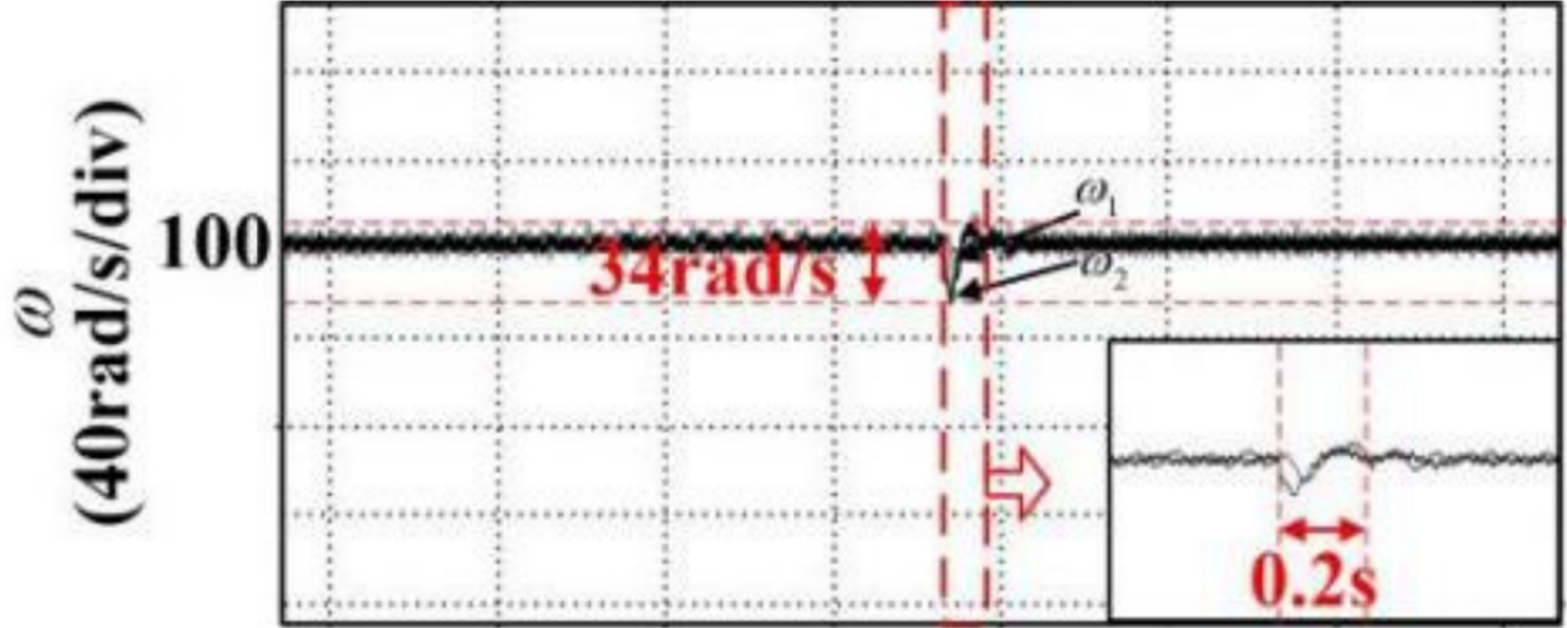
Velocity anti-disturbance fluctuation curves of the compared articles.

**Fig 33:**
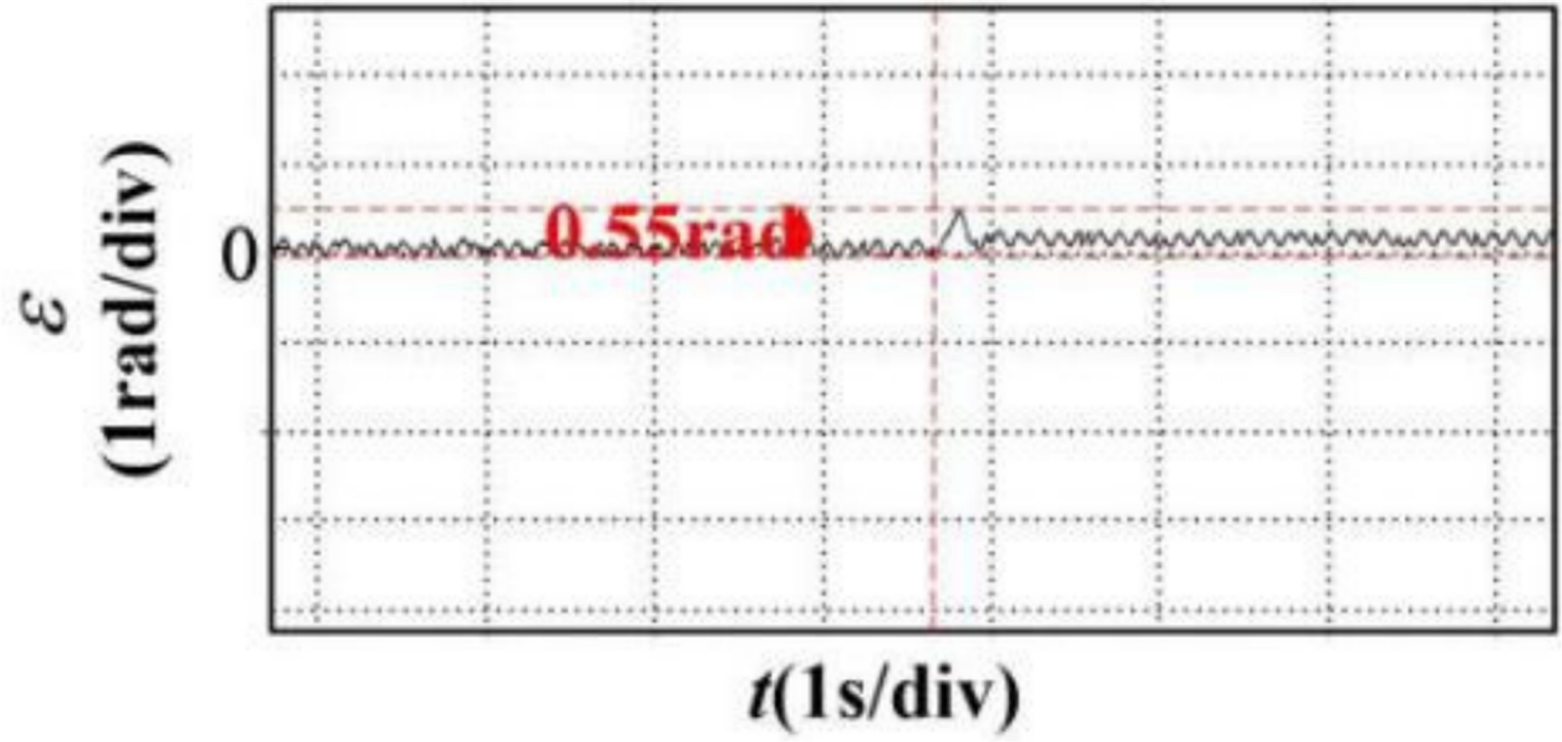
Position error immunity fluctuation curves of the compared articles.

**Fig 34:**
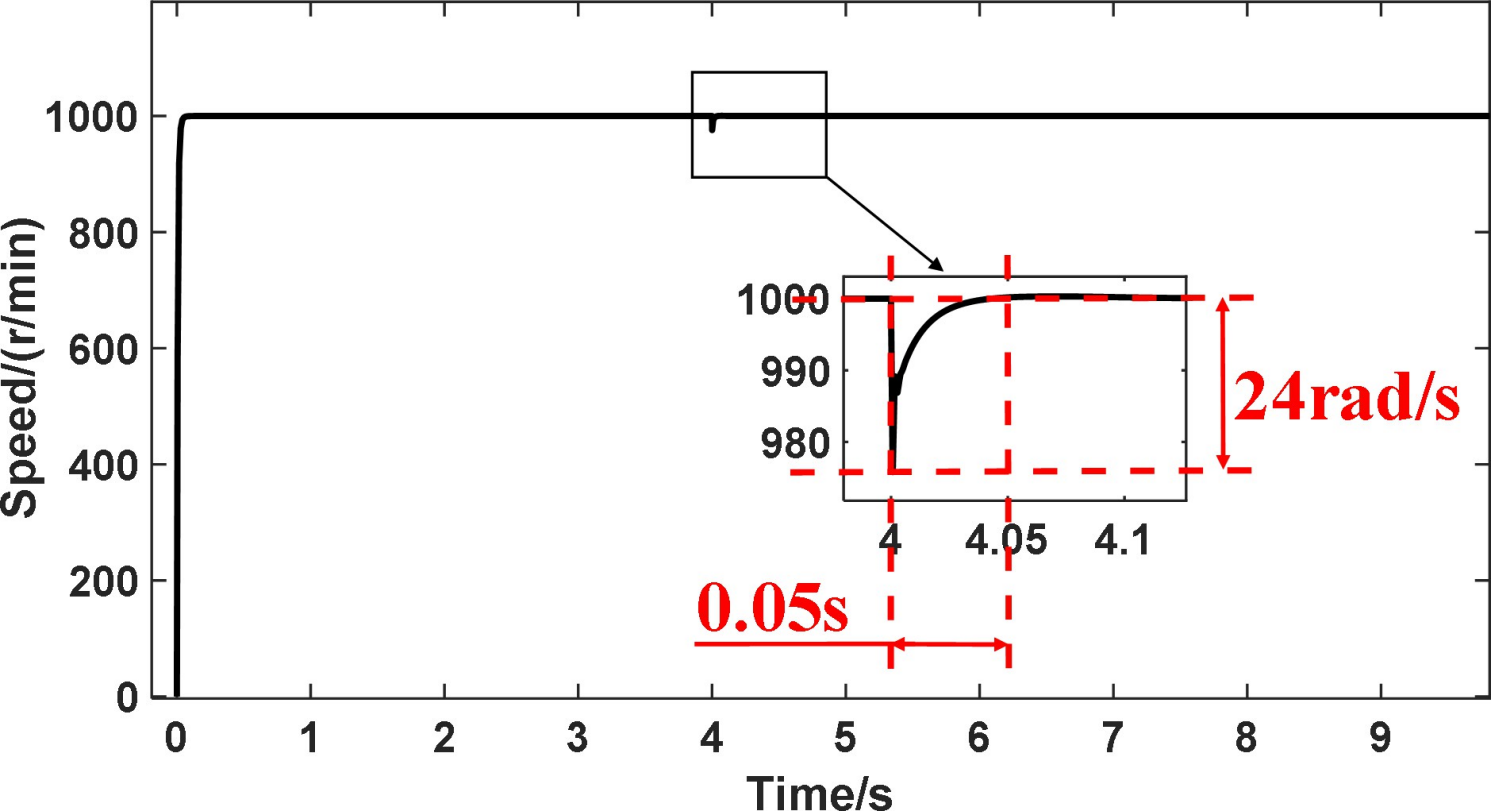
The velocity immunity curve is obtained in this paper.

**Fig 35:**
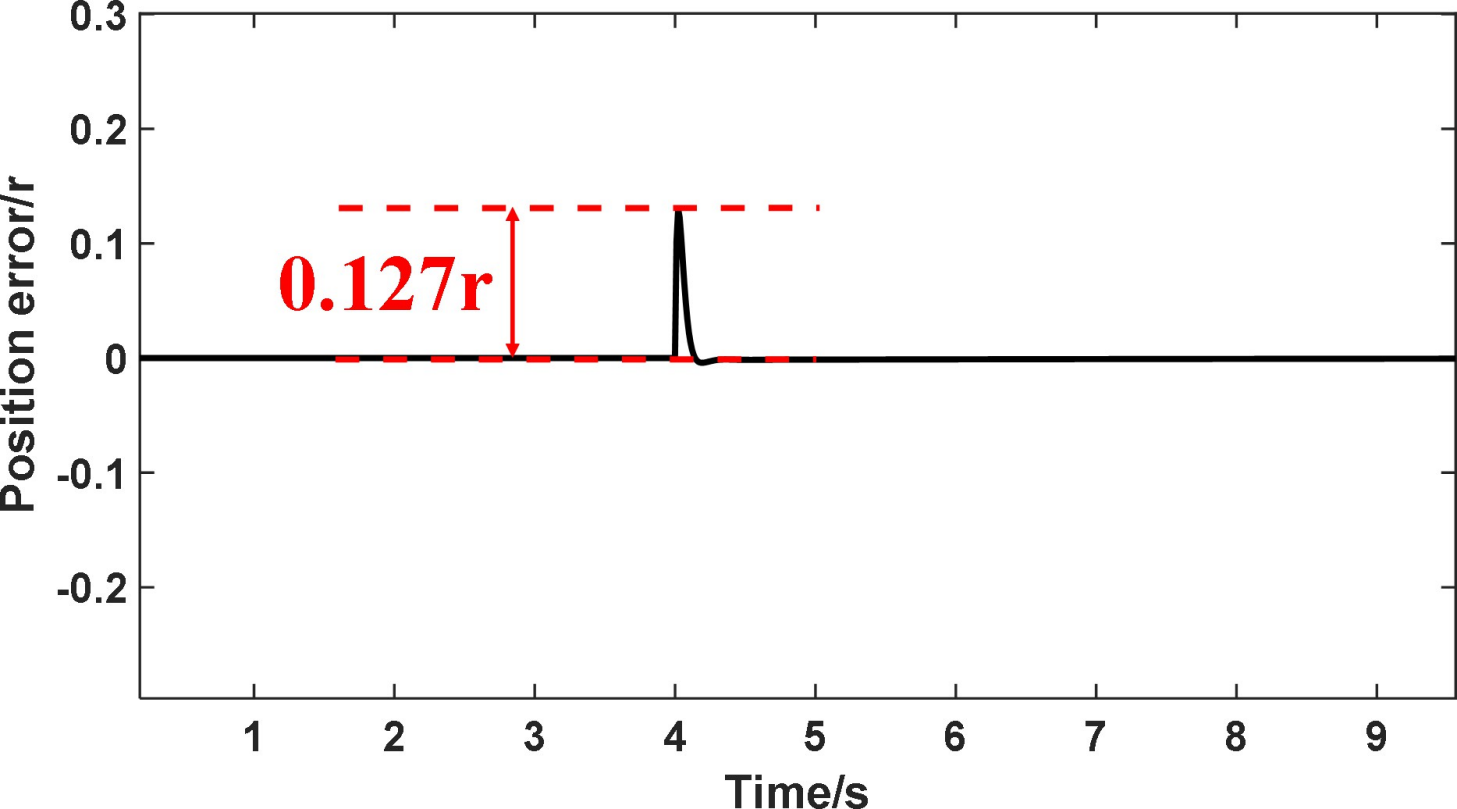
The position error immunity curve in this paper.

In the article [36], an anti-disturbance integrated position synchronization control strategy for a dual permanent magnet synchronous motor system is proposed for the problem that the position synchronization performance of the double permanent magnet synchronous motor servo system is vulnerable to parameter perturbation and load perturbation. Among them, in order to improve the synchronization performance of the system, a cross-coupled position synchronization compensator is added based on the overall mathematical model of the dual permanent magnet synchronous motor system and the third-order linear extended state observer, based on which a comparative analysis with this paper is carried out. Since the article [36] does not specify the additional disturbance’s significance, the motor motion with a disturbance torque of 6 Nm is selected for comparative data analysis in this paper. It can be seen from Figs 32 and 33 that the motor under the control of the strategy proposed in [36] has a fluctuation of 34 rad/s in speed, 0.2s in recovery time and 0.55 rad in displacement error after the disturbance, and there is jitter and vibration in both speed and position error without disruption; the simulation in this paper can be seen from Figs 34 and 35. It can be seen that the speed fluctuation size of the motor is 24 rad/s, the recovery time is 0.05s and no overshoot, the displacement fluctuation size is 0.127r. Neither the speed nor the displacement error produces jitter vibration. This paper’s research idea and method are worthy of reference and study.

## Conclusion

In this paper, an improved deviation-coupled multi-motor position synchronous control method based on non-singular fast terminal sliding mode control (NFTSMC) is proposed for the first time to address the problem of high-precision synchronous control of multi-motor positions Among them, NFTSMC can improve the dynamic performance of the motor It reduces the starting time of the motor, reduces the overshoot phenomenon and increases the robustness of the motor The improved deviation-coupled multi-motor synchronous control structure is based on the original two improvements, the first is to enhance the coupling between multi-motors, using PI gain module instead of the original fixed gain module in the compensator, after simulation and debugging to determine the parameters to achieve the purpose of coupling enhancement; the second in order to achieve multi-motor position synchronization, the motor output speed signal is integrated so that the output is position Secondly, in order to achieve multi-motor position synchronization, the motor output speed signal is integrated to make it output as position signal and fed back to the compensation to achieve position synchronization The simulation model of NFTSMC controller and multi-motor position synchronization control structure based on MATLAB/Simulink is established for simulation comparison, and the results show that the improved deviation-coupled multi-motor synchronization control method based on non-singular fast terminal sliding mode control can better control the multi-motor synchronous operation, and has short convergence time, fast transient response, steady-state response, and fast transient response compared with other control algorithms and multi-motor position synchronization control structures It has the advantages of short convergence time, fast transient response, small stationary error and high position tracking accuracy compared to other control algorithms and multi-motor position synchronization control structures, and it has higher reliability and solves the problems of unstable process. Control and inability to synchronize each motor’s positions with the highest precision in the industry.

## Supporting information

S1 Table FTSMC parameter settings. (XLSX)

S2 Table Maximum synchronization error between motors under three control algorithms. (XLSX)

S3 Table Motor parameter settings. (XLSX)

S4 Table NFTSMC controller parameter settings. (XLSX)

S5 Table README.md. (TXT)

S6 Table SMC parameter settings. (XLSX)

S7 Table Symbol naming table. (XLSX)

S8 Table Table of maximum synchronization error between motors at 800 r/min motor speed for three control methods. (XLSX)

S9 Table Table of maximum synchronization error between motors at 1000 r/min motor speed for three control methods. (XLSX)

S10 Table Table of maximum synchronization error between motors at 800 r/min motor speed for three control methods. (XLSX)
